# *Akkermansia muciniphila*–Driven ceRNA Networks Regulate Immune Modulation and Breast Cancer Progression

**DOI:** 10.1155/tbj/1416629

**Published:** 2026-05-26

**Authors:** Uma Chaudhary, Arya A. S., Mythili A.

**Affiliations:** ^1^ Department of Biotechnology, School of Biosciences and Technology, Vellore Institute of Technology (VIT), Vellore, 632014, Tamil Nadu, India, vit.ac.in; ^2^ Department of Sensor and Biomedical Technology, School of Electronics Engineering, Vellore Institute of Technology (VIT), Vellore, 632014, Tamil Nadu, India, vit.ac.in

**Keywords:** breast cancer, immune-regulation, next-generation probiotics, noncoding RNA

## Abstract

**Background:**

Microbiota‐derived metabolites are increasingly recognized as modulators of systemic immunity and cancer biology. This study investigates how a structurally distinct lipid from *Akkermansia muciniphila* influences immune transcriptional programs and their connection to breast cancer (BRCA)‐associated pathways.

**Methods:**

Donor‐adjusted reanalysis of PBMC RNA‐seq data was performed to identify lipid‐responsive transcriptional changes while minimizing interindividual variability. Differential expression was assessed across time points, followed by pathway enrichment and immune gene filtering. Immune cell composition was inferred using deconvolution analysis. Integration with The Cancer Genome Atlas (TCGA)–BRCA datasets enabled tumor immune infiltration profiling and network‐based identification of hub genes. ceRNA interactions were refined using correlation‐supported datasets and prognostic relevance was evaluated in TCGA and METABRIC cohorts.

**Results:**

Transcriptional variation was primarily driven by the treatment and exposure duration rather than donor effects. A biphasic immune response (IR) was observed, with early suppression followed by progressive activation. Lipid‐responsive genes significantly overlapped with BRCA immune signatures and were enriched in metabolic and stress‐related pathways. Immune deconvolution revealed shifts in macrophage polarization and cytotoxic cell populations. Network analysis identified key regulators, including ADIPOR1, KLF4, MYC, CXCL10, and ALDH1A1, linked to distinct immune infiltration patterns. ceRNA networks highlighted oncogenic and tumor‐suppressive miRNA interactions. A five‐gene signature demonstrated moderate prognostic value across cohorts.

**Conclusion:**

Microbial lipid signaling induces dynamic immune reprogramming that converges on tumor‐relevant pathways, suggesting a systemic immune‐mediated link between microbiota and BRCA progression, with potential implications for immune‐targeted therapeutic strategies.

## 1. Introduction

Breast cancer (BRCA) is the most prominent type of malignancy and a leading cause of cancer‐related mortality among women [1]. BRCA is highly heterogeneous, driven by varying histological subtypes and biological characteristics [2]. Although various conventional therapy methods are in use, recent studies have raised concerns about their effectiveness due to their nontargeted effects and side effects. Thus, probiotics and postbiotics have emerged immensely as potential biotherapeutics to treat a variety of diseases, including cancer [3–5]. The human body comprises a large population of symbiotic microorganisms, which are responsible for maintaining overall health. Some external and internal factors lead to the disruption of this microbiota equilibrium. This state of dysbiosis is often addressed by the administration of these biotherapeutics [6]. Recently, the field of biotherapeutics has seen the emergence of next‐generation probiotics (NGPs), which have become more evident as a potential biotherapeutic due to their specificity in the mode and target of action, safety, and their broad spectrum of action [7].

*Akkermansia muciniphila* is a potential NGP, an anaerobic, gram‐negative bacterium initially isolated from feces, which accounts for about 5% of the total intestinal microbiota [8]. Being a gut microbe, mucin, secreted by the goblet cells in the intestine, serves as its primary source of energy [9]. *A. muciniphila* has immunomodulatory properties by eliciting adaptive IRs by enhancing the IgA, IgG1, and antigen‐specific T‐cell responses [10]. The lipopolysaccharide (LPS) produced by *A. muciniphila* also influences the IRs by enhancing the production of IL‐8, IL‐6, and IL‐10 by stimulating peripheral blood mononuclear cells (PBMCs). They also enhance the production of TNF‐α through TLR4. Amuc_1100, an outer membrane protein associated with *A. muciniphila*, is an evident TLR2 activator, thereby inducing the production of IL‐1β, IL‐10, IL‐6, IL‐8, and also TNF‐α [11]. Hence, *A. muciniphila* influences the host immune system; rather than acting as an anti‐ or proinflammatory component, it maintains the gut equilibrium. Studies have shown that the comparatively reduced abundance of *A. muciniphila is* associated with certain tumor tissues. Patients having high concentrations of *A. muciniphila* exhibit an improved antitumor IR. These often involve the elevated CD8+ cytotoxic T cells, Treg cells, and cytokines like IL‐12 [12]. It also improves the efficiency of cancer immunotherapies and could also act as a biomarker for prognosis prediction [11]. A structurally unique lipid diacyl phosphatidylethanolamine with two branched chains (a15:0‐i15:0 PE) produced by *A. muciniphila* is known to have a certain immunomodulatory response with the aid of a toll‐like receptor TLR2‐TLR1 heterodimer [12, 13]. The effect of this bacterial lipid on human blood monocytes is described in the GEO dataset GSE199367.

While *A. muciniphila* has potent roles in eliciting cancer immunotherapy outcomes through immune modulation, its impact on BRCA remains unexplored. Most studies have focused on microbial associations in terms of probiotics, gut microbes, and host immune biomarkers, while the transcriptional relevance of defined bacterial components within an oncological context is yet to be addressed [14, 15]. Moreover, the contribution of noncoding RNAs, such as lncRNAs and miRNAs, as mediators of cross talk between tumor immune regulation and microbial‐derived components remains underexplored [16, 17]. A structured understanding of this interplay could provide deeper insights into the regulation of the breast tumor microenvironment by gut microbiota activities.

In this study, we hypothesized that exposure to the *A. muciniphila*‐derived lipid a15:0‐i15:0 PE [13] induces altered expression of BRCA‐related genes, potentially mediated or influenced by noncoding RNAs. This study is a retrospective computational observational study using secondary transcriptomic data with external cohort‐based survival validation. Importantly, we further focused on stage‐specific disease dynamics and the perimenopausal population (45–55 years), an area that has received limited attention, to uncover context‐dependent regulatory mechanisms. The identification of DEGs and the construction of an mRNA–ncRNA interaction network [18] may provide a better understanding of *A. muciniphila* and its bioactive lipid as adjunctive anticancer agents for BRCA therapies. To measure systemic immune modulation by microbial‐derived lipid exposure, PBMC transcriptomic changes were used here as a proxy. Since immune cells circulating in the blood are in constant communication with tumor tissues, systemic immune changes at this level could impact the recruitment, activation, and polarization of main immune populations such as macrophages, CD8+ T cells, natural killer (NK) cells, and regulatory T cells in the breast tumor microenvironment.

## 2. Materials and Methods

### 2.1. Transcriptomic Data Acquisition and Integrative Analysis

#### 2.1.1. Gene Expression Data Acquisition and Processing

The gene expression dataset GSE199367 was retrieved from the NCBI Gene Expression Omnibus (GEO) database [12]. This dataset comprises RNA‐seq profiles of monocytes isolated from human PBMCs derived from two independent donors. A total of 126 samples were included, consisting of untreated controls (positive and negative) and experimental groups treated with the lipid a15:0‐i15:0 PE, derived from *A. muciniphila.* Cells were treated with bacterial lipid fractions (Stim_A1, Stim_A2, Stim_A3), DMSO (negative control), LPS (positive control), Pam3CSK4, and FSL‐1 across three time points (2, 6, and 12 h), with three biological replicates per condition. The experimental design was balanced across donors, treatments, and time points. Clinical metadata such as age and sex were not available in the GEO submission as shown in Supporting Table S1 [12, 19].

#### 2.1.2. Differential Gene Expression Analysis

Differential expression analysis was performed using GEO2R, an online tool provided by NCBI GEO. GEO2R applies statistical modeling through the GEOquery and limma R packages from the Bioconductor project [19]. Comparisons were carried out between treated and control groups, and false positives were minimized using the Benjamini–Hochberg correction for multiple testing. Genes meeting the criteria of adjusted *p* value < 0.05 and |log2 fold change| ≥ 1 were considered differentially expressed genes (DEGs).

#### 2.1.3. Transcriptomic Data Normalization, Dimensionality Reduction, and Donor‐Adjusted Differential Expression Analysis

To address interdonor variability and further validate the robustness of the results, downstream transcriptomic analyses were conducted in R using the DESeq2 package on the raw count matrix [20, 21]. A multifactor design (∼donor + time + condition + time: condition) was employed to model donor effects, treatment time points, and their interactions. Before proceeding with the analysis, we removed genes that were barely expressed using the same statistical criteria. A variance‐stabilizing transformation (VST) was carried out on the count data for normalization and quality control purposes. PCA was then performed, and clustering on the hierarchical level was done using the pheatmap package on the distance metrics applied to variance‐stabilized expression data. Clustering was applied to both genes and samples. Basically, these analyses were quite insightful in determining sample clustering patterns, and they also convinced us that the main source of transcriptomic differences was the treatment and time point rather than donor identity. Besides that, no separate batch correction was implemented since donor effects were directly modeled in the design [21, 22].

#### 2.1.4. Integration With BRCA Expression Profiles

To identify genes relevant to BRCA, the DEGs obtained from GSE199367 were compared with expression profiles of BRCA available in the Gene Expression Profiling Interactive Analysis 2 (GEPIA2), which integrates RNA‐seq data from The Cancer Genome Atlas (TCGA) and the Genotype‐Tissue Expression (GTEx) project [23]. The overlapping genes between the datasets were identified using InteractiVenn, an online tool for generating Venn diagrams [24].

### 2.2. Probiotic Potential and Functional Annotation of *A. muciniphila*

The genomic characteristics of *A. muciniphila* (GCA_000020225.1) were assessed to evaluate its probiotic potential. ProbioMinServer was employed to calculate the Probiotic Potential Risk Score (PPRS), which integrates information on antibiotic resistance genes (ARGs), virulence factors (VFs), pathogenic genes (PGs), plasmids, and prophages [25]. To further annotate and explore the functional capacity of *A. muciniphila*, eggNOG‐mapper v2 was utilized to assign KEGG Orthology (KO) identifiers [26]. These KO identifiers were processed through the KEGG REST API for functional and enrichment analyses [27]. Pathways with a Benjamini–Hochberg‐adjusted false discovery rate (FDR) < 0.05 were considered significant. Probiotic‐relevant pathways were specifically examined using keywords related to vitamin biosynthesis, biofilm formation, and short‐chain fatty acid (SCFA) metabolism.

### 2.3. Immune‐Related Gene Filtering and Subtype Analysis

The overlapping DEGs (DE‐mRNAs) were further filtered using an immune‐related gene set associated with BRCA from the TISIDB database, a comprehensive resource for tumor–immune system interactions [28]. Gene symbols were batch‐queried to assess their immune relevance, and expression profiles were examined across the IR and molecular (MO) subtypes of BRCA. Candidate genes were prioritized based on the Kruskal–Wallis test, where a higher –log10(*p* value) in BRCA compared to other cancer types indicated stronger subtype‐specific immune regulation.

The STRING database was employed to construct a protein–protein interaction (PPI) network for the 30 differentially expressed immune‐responsive mRNAs identified in BRCA.

### 2.4. Functional Enrichment Analysis

Gene Ontology (GO) analysis of the common DE‐mRNAs was performed using the ShinyGO web server (2023 release). GO terms were categorized into three domains: biological process (BP), molecular function (MF), and cellular component (CC) [29]. Pathway enrichment analysis was carried out using the Enrichr platform with Reactome (2022), KEGG (2021), and WikiPathways (2023) databases. Significantly enriched terms and pathways were identified using a *p* value cutoff of < 0.05, and results were visualized as bar plots for interpretation [30].

### 2.5. Noncoding RNA Data Collection

To construct the regulatory networks, multiple classes of ncRNAs were retrieved, including miRNAs (microRNAs) and lncRNAs (long noncoding RNAs). miRNAs targeting the differentially expressed mRNAs (DE‐mRNAs) were collected from the miRNet 2.0, mirDIP 4.1, and miRTarBase databases [31–33]. lncRNAs interacting with DE‐mRNAs were obtained from LncExpDB and ENCORI (StarBase) [34, 35]. lncRNAs targeted by miRNAs were further extracted using miRNet 2.0 [31].

### 2.6. Network Construction and Subnetwork Identification

Cytoscape v3.10.2 was employed to construct and visualize the integrated regulatory network comprising mRNAs, miRNAs, and lncRNAs. Interaction datasets, including mRNA–miRNA, mRNA–lncRNA, and miRNA–lncRNA, were imported as CSV files and merged using the *Merge* tool. To identify densely connected modules, the MCODE plugin was applied with the following parameters: degree cutoff = 2, node score cutoff = 0.2, k‐core = 2, and maximum depth = 100. The top‐ranked subnetwork (Cluster 1), representing the integrated mRNA–miRNA–lncRNA module, was identified with a score of 6.356. MCODE analysis was applied to identify densely connected regulatory modules, as such clusters often represent functionally significant biological interactions. Further prioritization of key interactors and hub regulators within these modules will be performed using CytoHubba and MClique to refine the identification of critical nodes in the network [36].

### 2.7. Gene Expression Analysis of mRNAs, miRNAs, and lncRNAs

Following the identification of hub RNAs, expression analysis was performed using UALCAN, a comprehensive web resource for exploring cancer omics data. The TCGA‐BRCA dataset, complemented with GTEx normal tissue data, was employed to evaluate the expression profiles of the selected hub mRNAs, miRNAs, and lncRNAs. Special emphasis was placed on analyzing expression patterns across different cancer stages in perimenopausal women (aged 45–55 years). The statistical significance of expression differences was determined, and relevant findings were further integrated with survival analysis outcomes to assess their prognostic impact [37].

### 2.8. Correlation‐Based Filtering of miRNA–mRNA Interactions (ENCORI)

mRNA–miRNA interaction data of a set of selected mRNAs were extracted from the ENCORI platform. Both experimentally verified and computationally predicted miRNA‐target interactions were assembled for five candidate genes (ADIPOR1, ALDH1A1, KLF4, MYC, and CXCL10). Correlation analysis offered by ENCORI was used as a first step to ensure biological relevance following the ceRNA hypothesis. Network obtained from MCODE gone under correlation‐based filtering to find those interactions displaying significant statistical differences (p < 0.05) and showing a negative correlation between miRNAs and their target mRNAs, which is a hallmark of miRNA‐mediated repression, were kept. Furthermore, to make the analysis more reliable, very weak correlations (|*r*| < 0.1) were also removed. These high‐confidence interactions served as a basis for further interpretation of regulatory relationships.

### 2.9. Immune Deconvolution and Immune Infiltration Correlation

#### 2.9.1. Immune Infiltration and Gene Expression Analysis

The TIMER2.0 database (Tumor Immune Estimation Resource) was utilized to evaluate the immune relevance of the five‐gene signature (ADIPOR1, CXCL10, ALDH1A1, MYC, and KLF4) in BRCA. The *Gene_DE* module was applied to compare differential expression between tumor and adjacent normal tissues, with significance assessed using the Wilcoxon test. The *Gene_Corr* module was employed to investigate pairwise correlations among the five genes, with correlation strength determined by purity‐adjusted Spearman’s rho values.

In addition, an immune infiltration analysis was carried out through the CIBERSORT algorithm, within the TIMER2.0 web server, to figure out how hub gene expressions are connected to the infiltration levels of immune cells in the TCGA‐BRCA cohort. Spearman correlation analysis was used for assessing the relation between gene expression and immune cell abundance, with a significance level set at *p* < 0.05. Both statistically significant gene‐immune cell relations were limited, and the association’s direction was found by using the correlation coefficient (rho). The immune cell populations examined were macrophage subsets (M0, M1, and M2), NK cells, CD4 T‐cell subsets, CD8 T cells, and regulatory T cells (Tregs). TIMER2.0 was selected for this analysis as it systematically integrates immune deconvolution methods across TCGA cancers, enabling robust exploration of gene expression patterns in relation to the tumor immune microenvironment [38].

#### 2.9.2. Immune Deconvolution of PBMC Transcriptomes (GSE199367)

Immune deconvolution of PBMC transcriptomic profiles was performed using the CIBERSORT method via the TIMER2. 0 platform. The input was gene expression data normalized to TPM with HGNC gene symbols in the gene‐by‐sample format. This method uses a standardized leukocyte gene signature matrix to infer the relative proportions of 22 immune cell types. In order to focus on the major immune cells for further analysis, macrophage subsets (M0, M1, and M2), NK cells, CD4+ T cell subsets, CD8+ T cells, and Tregs were mainly considered. Treatment conditions (DMSO, LPS FSL1, Pam3CSK4, and stimulations) were used for grouping samples, and immune cell fractions were then compared between groups with graphical visualization. Such analysis shows how the different treatments impact changes in the immune cell composition systemically.

### 2.10. Prognostic Model and mRNA‐Based Survival

#### 2.10.1. Genomic Alteration and Clinical Association Analysis

The cBioPortal platform was employed to assess genomic alterations in the selected five‐gene signature (ADIPOR1, CXCL10, ALDH1A1, MYC, and KLF4) using multiple BRCA datasets, including METABRIC, TCGA PanCancer Atlas, the Metastatic Breast Cancer Project, and the FUSCC TNBC cohort. OncoPrint analysis was performed to visualize the frequency and types of genomic alterations across patient samples. Clinical distribution analysis and cross‐cancer comparison were made to evaluate the heterogeneity of these alterations. Additionally, survival analysis was carried out to determine the prognostic relevance of the five‐gene signature in BRCA [39].

#### 2.10.2. Transcriptome‐Based Survival and Prognostic Model Construction

mRNA expression, as well as clinical information of BRCA, was derived from the TCGA‐BRCA dataset (training cohort) and the METABRIC dataset (validation cohort). Raw gene expression data were initially processed, log‐transformed if necessary, and then synchronized with clinical data in order to maintain the integrity of the sample comparisons. Five potential marker genes (ADIPOR1, ALDH1A1, KLF4, MYC, and CXCL10) identified as a result of network analysis were the basis for the development of a predictive model.

A multivariate Cox proportional hazards regression model was built using the TCGA data, and then, each patient’s risk score was computed by summing the product of gene expression levels and their respective regression coefficients (risk score = sum (coefficient × gene expression). Using the median risk score as the cutoff, patients were divided into high‐ and low‐risk groups. Survival probabilities were compared between risk groups by the Kaplan–Meier survival analysis and log‐rank tests. To measure model performance, concordance index (C‐index), time‐dependent receiver operating characteristic (ROC) curves at different time points, and calibration curves showing the relationship between predicted and observed survival probabilities were used. In addition, the prognostic model was validated on the independent METABRIC dataset using the same risk score calculation. To evaluate clinical relevance, the study first divided patients into age groups (< 45, 45–55, and > 55 years) as a rough indicator of menopausal status and then performed survival analysis for each subgroup.

## 3. Results

### 3.1. DEGs‐Based Analysis

#### 3.1.1. Identification of DEGs

GEO2R analysis identified 5320, 5929, and 6473 DEGs at 2, 6, and 12 h of a15:0‐i15:0 PE treatment, respectively, compared to controls. A total of 126 RNA‐seq samples were included in the analysis. The dataset comprised two independent donors, seven treatment conditions, and three time points (2, 6, and 12 h), each with triplicate measurements. The detailed distribution of samples is summarized in Supporting Table S1. Box plots confirmed data normalization and reliability, while volcano plots illustrated distinct expression dynamics. At 2 h, downregulated transcripts were predominant, suggesting an early suppressive response. By 6 h, a transcriptional shift was observed, with a marked increase in upregulated genes (log2FC ∼4–10). At 12 h, upregulated transcripts became dominant, highlighting a robust activation phase. These results demonstrate the time‐dependent immunomodulatory potential of the probiotic‐derived lipid, shifting from suppression to activation of gene expression (Figures 1(a), 1(b), 1(c), 1(d), 1(e), and 1(f)). GEO2R was used for initial DEG detection, and donor‐adjusted DESeq2 analysis confirmed the robustness of these findings.

FIGURE 1Illustration of volcano plots depicting the differential gene expression in PBMC on exposure to a15:0‐i15:0 PE extracts for (a) 2 h, (b) 6 h, and (c) 12 h compared to negative controls. Boxplots demonstrating the log10‐normalized RNA‐seq counts distribution of a15:0‐i15:0 PE‐treated PBMC and negative control at (d) 2 h, (e) 6 h, and (f) 12 h. The cross talk between DEGs identified in a15:0‐i15:0 PE‐treated PBMC and breast cancer (BRCA) gene sets across (g) 2 h, (h) 6 h, and (i) 12 h time points. (j) Illustration of overlap between differentially expressed genes (DEGs) from GSE199367 and immune‐associated genes from the TISIDB database.

**Figure:**
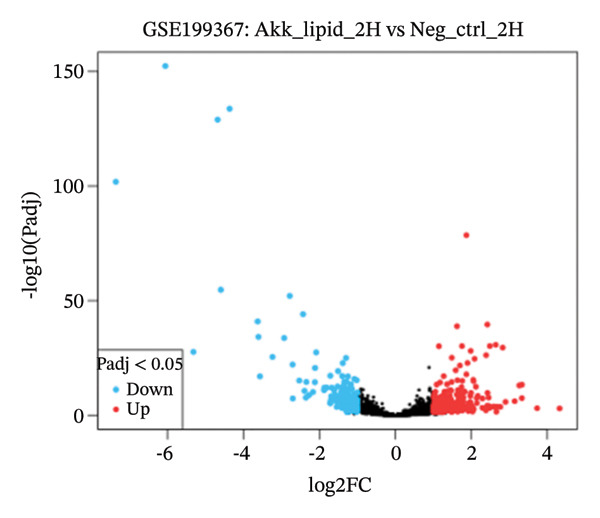
(a)

**Figure:**
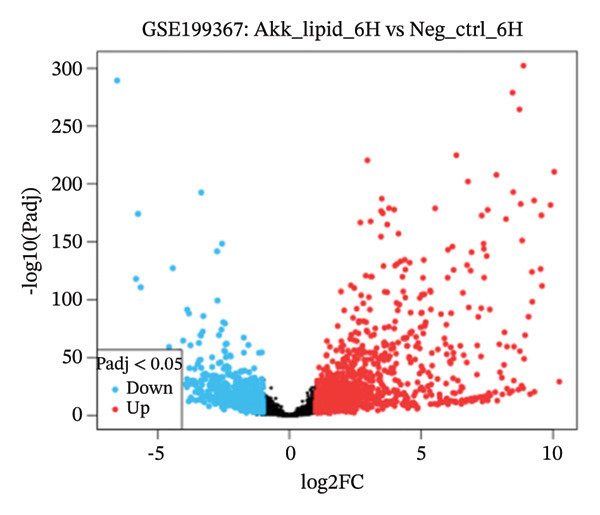
(b)

**Figure:**
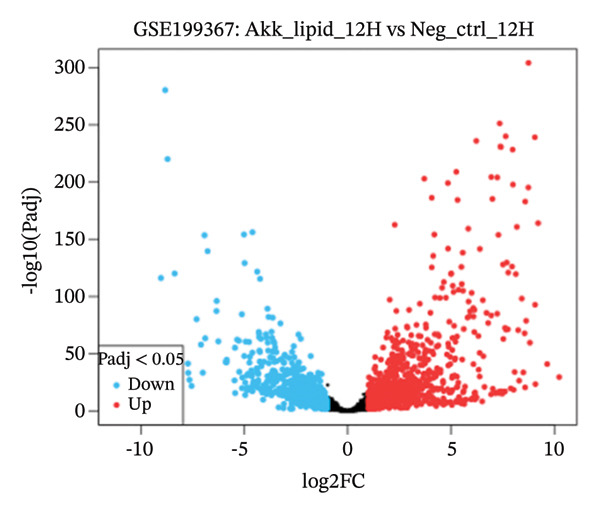
(c)

**Figure:**
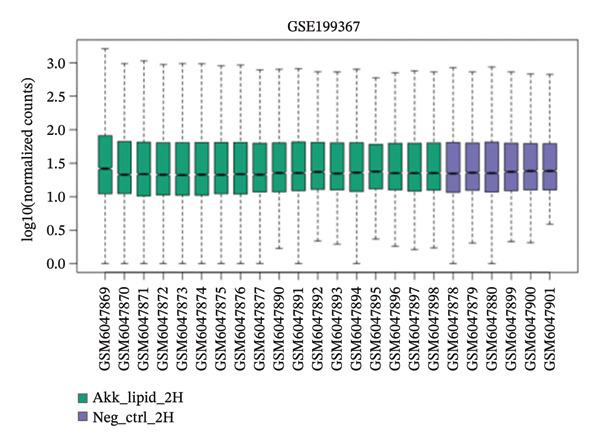
(d)

**Figure:**
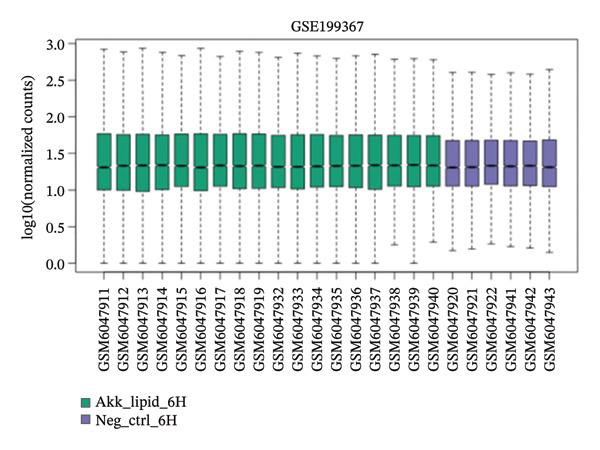
(e)

**Figure:**
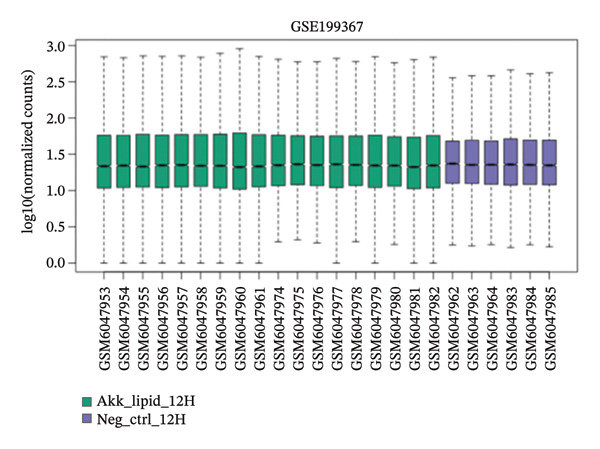
(f)

**Figure:**
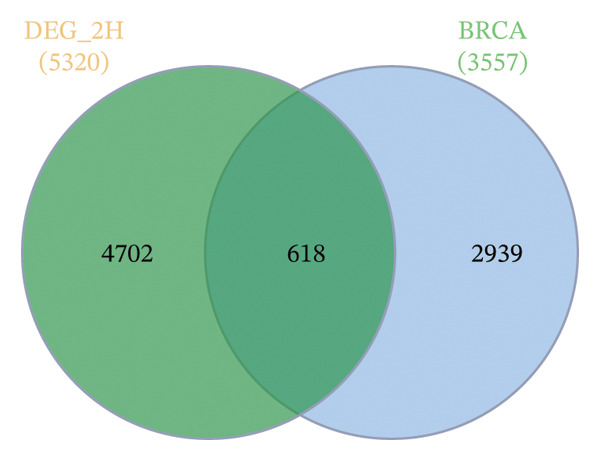
(g)

**Figure:**
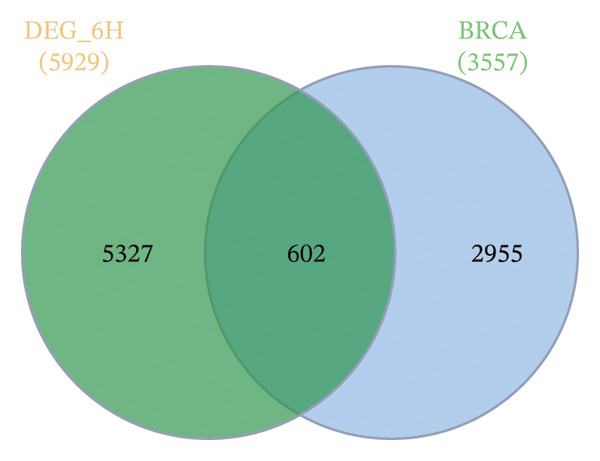
(h)

**Figure:**
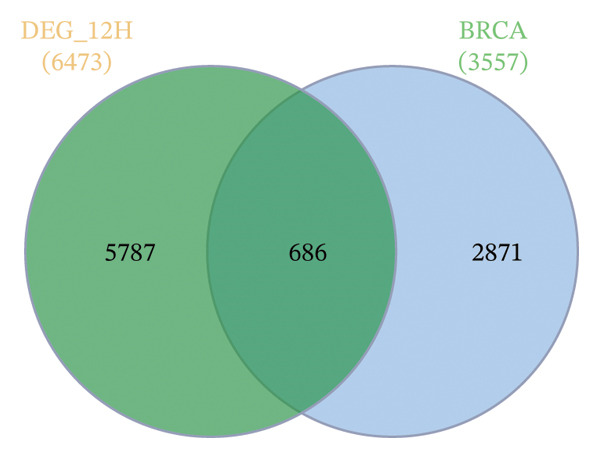
(i)

**Figure:**
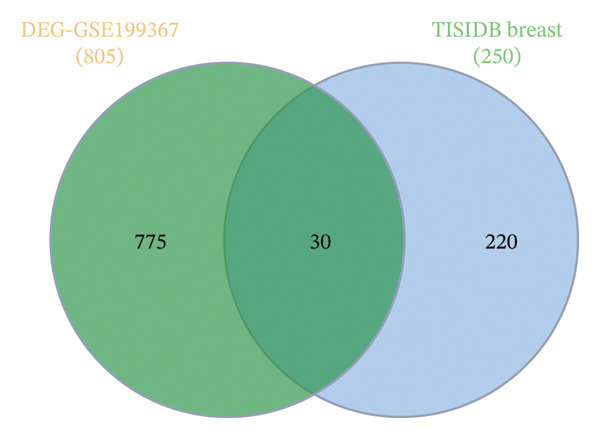
(j)

#### 3.1.2. DEGs and Donor Variability

**FIGURE 3:**
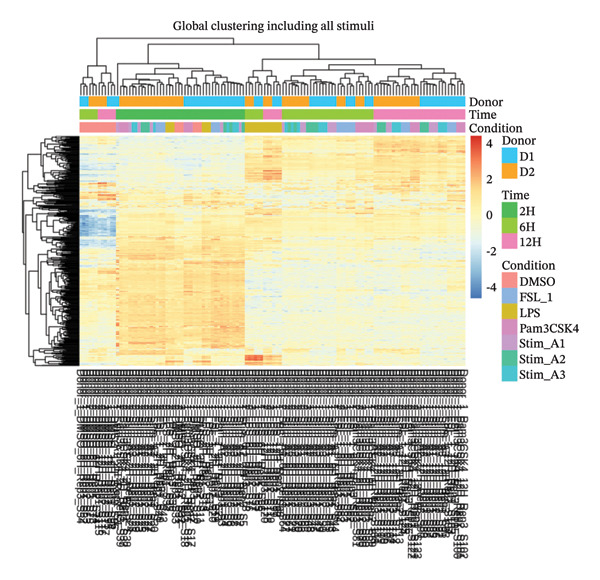
Global hierarchical clustering of all stimuli. Heatmap of differentially expressed genes shows sample clustering by donor, time point, and stimulation condition, indicating distinct transcriptional signatures across experimental groups.

Principal component analysis (PCA) of variance‐stabilized data demonstrated clear clustering of samples based on treatment conditions in Figure 2(b), with no evident segregation by the donor in Figure 2(a), indicating that interindividual variability did not confound the analysis. Among treatments, LPS induced the most pronounced transcriptional shift, while other stimuli exhibited distinct but partially overlapping expression patterns. Differential expression analysis revealed time‐dependent responses, with an increasing number of DEGs at later time points, reflecting progressive transcriptional modulation. Hierarchical clustering further confirmed condition‐ and time‐specific expression patterns independent of donor effects, shown in Figure 3. Additionally, a subset of consistently DEGs across all time points was identified, representing conserved transcriptional signatures associated with lipid‐induced IRs.

FIGURE 2Principal component analysis of gene expression profiles. (a) PCA plots show clear separation of samples between D1 and D2 groups, and (b) among different treatment conditions, indicating distinct global transcriptional responses.

**Figure:**
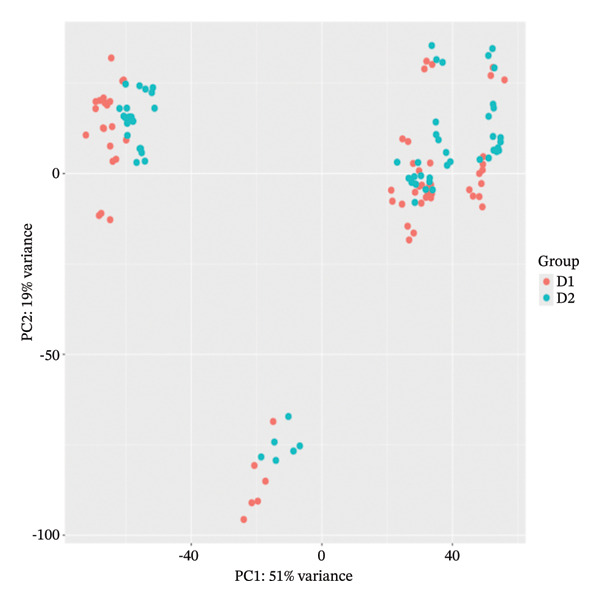
(a)

**Figure:**
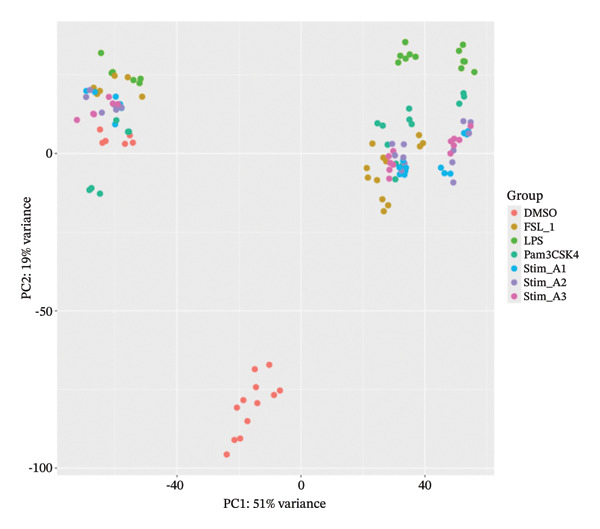
(b)

### 3.2. Interrelationship Between DEGs and BRCA Genes

A comparison with BRCA datasets (GEPIA2) revealed consistent overlap between a15:0‐i15:0 PE‐induced DEGs and BRCA‐associated genes (refer to tumor vs normal DEGs from GEPIA2/TCGA–GTEx). Specifically, 618 (11.62%) genes overlapped at 2 h, 602 (10.15%) at 6 h, and 686 (10.60%) at 12 h. The increasing proportion of overlap at later time points suggests that lipid‐induced transcriptional changes progressively converge with cancer‐related pathways. These findings imply a potential mechanistic link between host immune modulation and BRCA‐associated gene regulation (Figure 1(g), 1(h), and 1(i)).

### 3.3. Genomic Profiling and Probiotic Functional Potential of *A. muciniphila*

**FIGURE 4:**
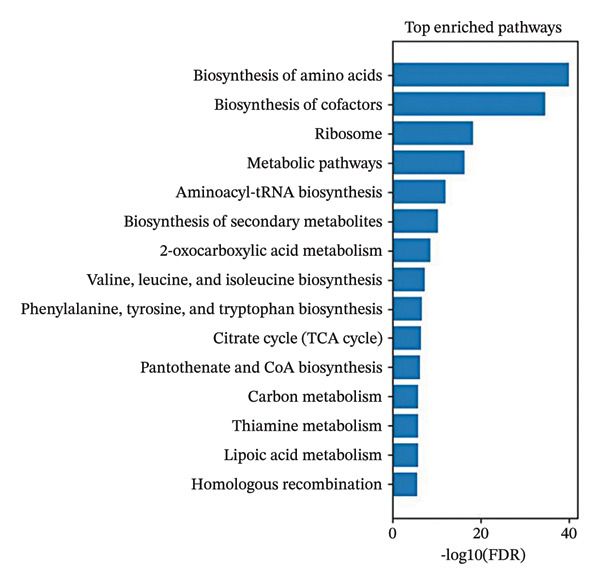
Genomic profiling of *A. muciniphila* highlighting functional enriched pathways.

The genomic analysis of *A. muciniphila* (GCA_000020225.1) demonstrates a strong probiotic safety profile, with ProbioMinServer reporting a low PPRS—no detectable ARGs, VFs, or plasmids were found, indicating a minimal risk of pathogenicity. Functional annotation further highlighted significant enrichment in primary metabolic, biosynthetic, and vitamin pathways (FDR < 0.05), notably including amino acid metabolism (34.9% coverage), amino‐acyl tRNA synthesis (37.9%), and protein synthesis (26.4%). The organism also showed robust biosynthetic potential for several vitamins (pantothenate, thiamine, folate, riboflavin, and biotin), exopolysaccharides, and SCFAs such as propionate and butanoate. Additionally, genomic evidence for regulatory mechanisms—including two‐component systems and quorum sensing—suggests enhanced adaptability and colonization capacity in the host environment (Figure 4).

### 3.4. Immune‐Related Gene Prioritization and Selection

Using the TISIDB database to filter for immune relevance, 30 key genes were identified by intersecting the 805 DEGs from a15:0‐i15:0 PE‐treated PBMCs with 250 BRCA‐associated immune genes. These immune‐related 30 candidates were selected for subsequent functional enrichment and network analyses, highlighting their potential roles in tumor–immune interactions in BRCA (Figure 1(j)).

### 3.5. Functional Enrichment Analysis of Immune‐Related Genes

Functional enrichment analysis of the 30 prioritized immune‐related genes revealed significant associations across WikiPathways, Reactome, and KEGG databases. Figure 5(a) shows key enriched pathways included adipocytokine signaling, AMPK signaling, longevity regulation, pluripotency of stem cells, and peptide hormone metabolism. Notably, pathways involved in cell differentiation, energy metabolism, wound healing, and signaling mechanisms relevant to cancer and immune regulation were prominently represented, indicating a multifaceted biological impact of these genes. Figure 5(b) shows GO term enrichment further categorized functional relevance across three domains: MF: Genes were enriched for cytokine binding, chemokine receptor interaction, growth factor receptor activity, and lipid binding functions. C C: Top locations included the extracellular space, plasma membrane, membrane‐bounded organelles, secretory granules, and vesicles, highlighting secretory and signaling roles. B P: There was significant enrichment for regulation of inflammatory response, general and defense responses to stress, oxygen‐containing compounds, and external stimuli, underscoring a major role in host immune modulation and inflammation. Collectively, these results demonstrate that the selected immune‐related genes are functionally specialized in pathways that govern cell signaling, metabolic adaptation, IRs, and tumor biology, aligning with the study’s focus on immunomodulatory mechanisms and cancer relevance.

FIGURE 5(a) Pathway enrichment of dysregulated genes in breast cancer across WikiPathways, Reactome, and KEGG databases. (b) Gene Ontology classification showing enriched molecular functions, cellular components, and biological processes.

**Figure:**
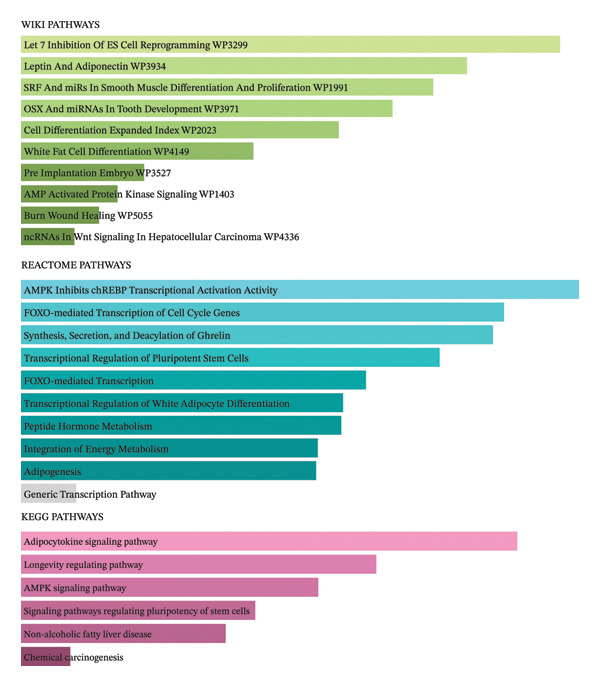
(a)

**Figure:**
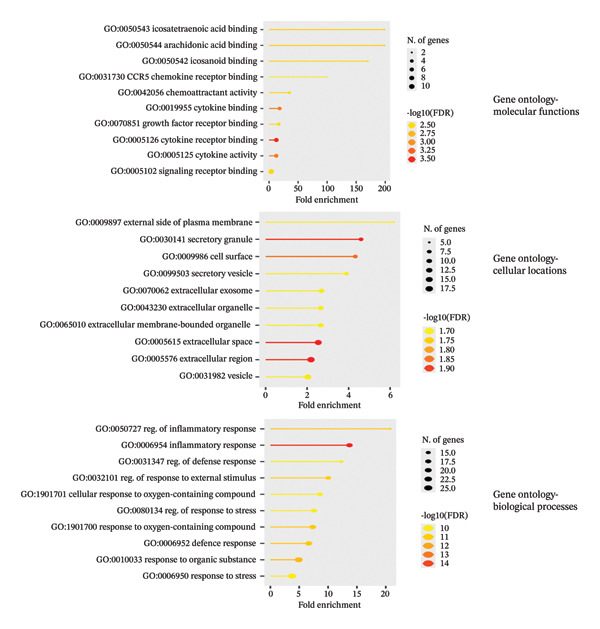
(b)

### 3.6. Construction of the ceRNA Network and MCODE‐Based Subnetwork Identification

The STRING‐derived PPI network of the 30 prioritized immune‐related genes, illustrating their functional connectivity and potential cooperative roles in BRCA progression, is depicted in Figure 6(a).

FIGURE 6(a) Protein–protein interaction (PPI) network of proteins encoded by 30 dysregulated genes. (b and c) Clustered subnetworks identified in Cytoscape.

**Figure:**
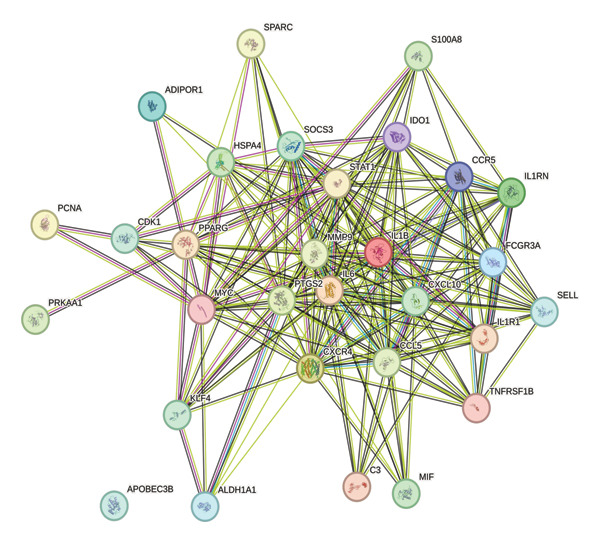
(a)

**Figure:**
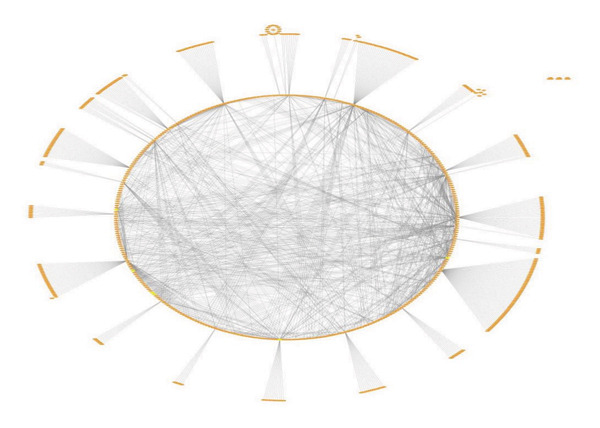
(b)

**Figure:**
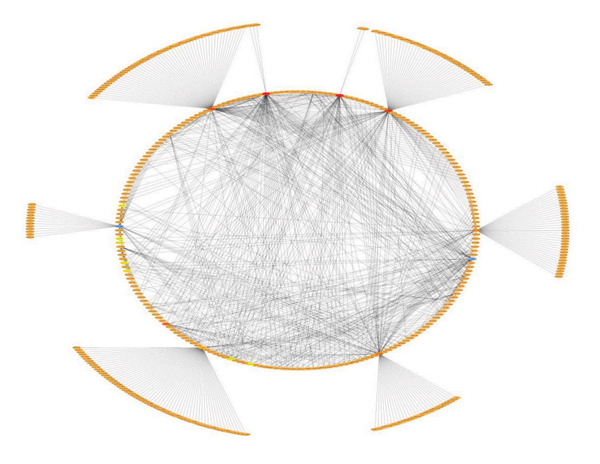
(c)

**FIGURE 7:**
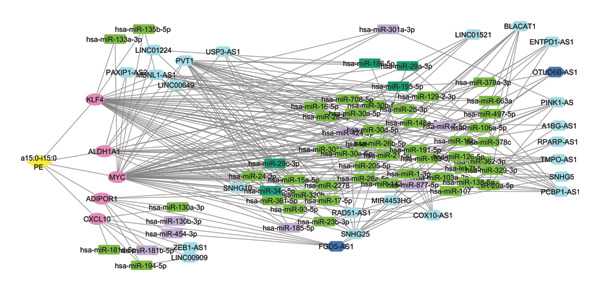
Top‐ranked gene cluster identified using the MCODE plugin in Cytoscape. The highly connected subcluster derived from the final cluster, representing genes targeted by the *Akkermansia* lipid. Yellow diamond—lipid, pink ellipse—mRNAs, green rectangle—miRNAs, turquoise rectangle—downregulated miRNAs, purple rectangle—upregulated miRNAs, and light blue hexagon—lncRNAs.

An integrated ceRNA network comprising mRNAs, miRNAs, and lncRNAs was constructed in Cytoscape, resulting in a merged network of 704 nodes and 1480 edges. Interaction patterns revealed mRNA–miRNA, mRNA–lncRNA, miRNA–lncRNA interactions (Figures 6(b) and 6(c)). MCODE analysis of the merged network identified Cluster 1 as the top‐ranked subnetwork (score = 6.356), consisting of 90 nodes and 268 edges, (Figure 7).

This module highlighted the central involvement of five mRNAs (ADIPOR1, ALDH1A1, MYC, KLF4, and CXCL10) linked to multiple noncoding RNAs. Additional subnetworks and triads generated using MClique further emphasized crucial relationships among mRNAs and ncRNAs, as depicted in Supporting Figure S2.

### 3.7. Topological Analysis Reveals Central Hub Genes and ncRNAs

Topological analysis using CytoHubba was performed to prioritize hub nodes within the interaction network by applying multiple centrality algorithms, including betweenness, degree, closeness, MCC, and EPC. The analysis revealed seven nodes that consistently ranked among the top candidates across several algorithms, underscoring their central role in maintaining network stability. These hub regulators—ADIPOR1, ALDH1A1, KLF4, and MYC—were repeatedly prioritized across different centrality measures, as shown in Supporting Figure S1. The detailed rankings for each algorithm are provided in Supporting Table S2.

### 3.8. Prognostic and Correlation‐Validated Hub RNA Interactions Identified by UALCAN and ENCORI Analyses

UALCAN‐based expression and survival analyses were performed to evaluate the clinical relevance of the hub RNAs. Among the top network candidates, several miRNAs demonstrated significant prognostic associations. Oncogenic/poor prognosis miRNAs included miR‐130b‐3p, miR‐454‐3p, miR‐181b‐5p, miR‐301b‐3p, miR‐877‐5p, miR‐424‐5p, miR‐185‐5p, and miR‐7‐5p, all of which were upregulated in BRCA. In contrast, tumor‐suppressive/favorable prognosis RNAs such as miR‐34c‐5p, miR‐186‐5p, miR‐29a‐3p, miR‐29c‐3p, miR‐195‐5p, FGD5‐AS1, and OTUD6B‐AS1 were downregulated. The analysis has been refined to include only statistically significant and correlation‐supported interactions. These findings highlight a subset of hub RNAs with stage, age, and race‐specific expression patterns that may serve as potential prognostic biomarkers in BRCA (Supporting Table S3).

Correlation‐based filtering of ENCORI‐derived interactions identified a subset of biologically consistent miRNA–mRNA pairs. The complete list of correlation‐based filtering interactions of mRNAs, miRNAs, and lncRNAs is provided in Supporting Table S4. Among these, several strong negative correlations were observed, suggesting potential post‐transcriptional regulation. Notably, KLF4 demonstrated multiple significant interactions, including with hsa‐miR‐301b‐3p (*r* = −0.23, *p* < 0.001), hsa‐miR‐15b‐5p (*r* = −0.23, *p* < 0.001), and hsa‐miR‐185‐5p (*r* = −0.179, *p* < 0.001). Similarly, ALDH1A1 showed a strong inverse association with hsa‐miR‐301b‐3p (*r* = −0.207, *p* < 0.001), while ADIPOR1 was negatively correlated with hsa‐miR‐130a‐3p (*r* = −0.251, *p* < 0.001), as illustrated in Supporting Figure S4. These interactions are consistent with the expected inhibitory role of miRNAs and highlight key regulatory axes that may contribute to transcriptional modulation in TNBC. In contrast, several predicted interactions displaying positive or weak correlations were excluded, reinforcing the importance of correlation‐based validation.

### 3.9. TIMER2.0 Analysis of the Five‐Gene Panel

#### 3.9.1. Gene Correlation Analysis

The TIMER Gene_Corr module revealed significant positive correlations among the five‐gene panel in BRCA and its MO subtypes. Notably, ADIPOR1 correlated strongly with KLF4 in BRCA, while CXCL10 displayed subtype‐specific positive correlations with ALDH1A1 and MYC in BRCA‐basal, and with ALDH1A1 in BRCA‐Her2, BRCA‐LumA, and BRCA‐LumB. Similarly, KLF4 was positively associated with ADIPOR1 and ALDH1A1 in BRCA and BRCA‐LumA, and with ALDH1A1 in BRCA‐LumB. In addition, MYC showed positive correlations with CXCL10 in BRCA‐basal and with ALDH1A1 in BRCA‐LumA. These results highlight subtype‐dependent coregulatory patterns, suggesting immune–oncogenic interactions within the tumor microenvironment, as shown in Supporting Figure S5.

#### 3.9.2. Gene Differential Expression Analysis

The TIMER Gene_DE module demonstrated distinct expression profiles of the five‐gene panel in BRCA. ADIPOR1 and CXCL10 were significantly upregulated in tumor tissues compared to normal tissues, supporting their potential oncogenic or immunomodulatory roles. Conversely, ALDH1A1, KLF4, and MYC were downregulated in tumors relative to normal samples, indicating loss of protective or regulatory functions during tumorigenesis. All differences were statistically significant (Wilcoxon test), underscoring the biological relevance of this gene set in BRCA, as shown in Supporting Figure S6.

#### 3.9.3. Immune Deconvolution of PBMC Transcriptomic Profiles

**FIGURE 8:**
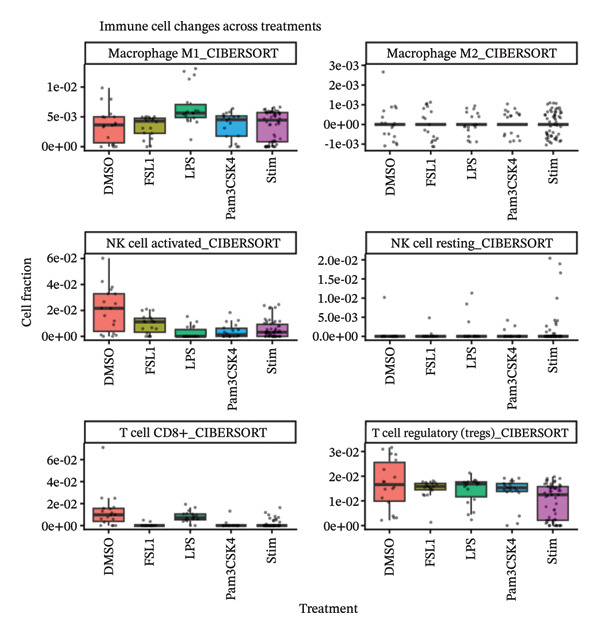
Deconvolution analysis of GSE199367. CIBERSORT‐derived fractions of macrophage M1/M2, activated and resting NK cells, CD8+ T cells, and regulatory T cells across treatments highlight stimulus‐dependent remodeling of the immune cell landscape.

CIBERSORT analysis of immune cells in PBMC transcriptome showed that certain treatments lead to different changes in immune composition; this deconvolution analysis of GSE199367 is shown in Figure 8. LPS stimulation strongly raised proinflammatory M1 macrophages proportion, revealing intensive immune activation. On the other hand, lipid‐related treatments, namely, Pam3CSK4 and FSL1, were linked to decreased amounts of activated NK cells and CD8+ T cells, thus implying the downregulation of cytotoxic IRs. M2 macrophages were found to be low in all samples, which signifies that the induction of anti‐inflammatory polarization was very limited. Tregs showed a bit of fluctuation but without any particular direction in the different treatments. In summary, the results indicate that lipids as stimuli change systemic immunity very much the macrophage polarization and cytotoxic immune cell populations, in particular. These observations support the hypothesis that microbiota‐derived lipid stimuli can reshape systemic immune cell composition, particularly macrophage polarization and cytotoxic populations, which may in turn influence the tumor immune microenvironment.

#### 3.9.4. Immune Infiltration Patterns of Hub Genes in BRCA

Immune infiltration analysis showed different relationships of hub gene expression with immune cell populations in BRCA (Supporting Figure S3). ADIPOR1 expression was found to be most strongly correlated with M2 macrophages and resting CD4+ memory T cells and was negatively correlated with M0 macrophages, activated NK cells, CD8+ T cells, and Tregs. This pattern is consistent with an overall immunosuppressive association. ALDH1A1 was positively linked to M1 macrophages, resting CD4+ memory T cells, and CD8+ T cells but showed negative correlations with M0 macrophages, resting NK cells, and Tregs. This suggests a mixed immunomodulatory profile. CXCL10 showed a very strong immune‐activating pattern; most of the immune cells were positively correlated: M1 macrophages, activated NK cells, CD4+ T‐cell subsets, CD8+ T cells, and Tregs, whereas it exhibited negative associations for M2 macrophages and resting NK cells. The level of KLF4 expression correlated positively with resting CD4+ memory T cells but appeared negatively associated with M0 macrophages, activated NK cells, activated CD4+ T cells, and Tregs. It is likely that this is a factor in immune regulation. MYC exhibited positive correlations with M1 macrophages, activated CD4+ T cells, and CD8+ T cells, while it was negatively correlated with M2 macrophages and Tregs, supporting its participation in modulating both immune activation and suppression pathways. Overall, these findings highlight consistent involvement of macrophage polarization and T‐cell dynamics across hub genes, suggesting their potential role in shaping the tumor immune microenvironment in BRCA.

### 3.10. Genomic Landscape and Survival Impact of the Five‐Gene Signature in BRCA

#### 3.10.1. Genomic Alterations of the Five‐Gene Signature

OncoPrint analysis revealed heterogeneous alteration patterns across the five‐gene signature (ADIPOR1, CXCL10, ALDH1A1, MYC, and KLF4) in BRCA cohorts. Among them, ADIPOR1 (20%) and MYC (20%) displayed the highest alteration frequencies, with MYC primarily exhibiting amplifications, while CXCL10 (2.3%), ALDH1A1 (1.4%), and KLF4 (1.2%) showed comparatively low alteration rates (Supporting Figure S7).

#### 3.10.2. Transcriptome‐Based Prognostic Model Based on the Five‐Gene Signature

A five‐gene risk score model was constructed in the TCGA‐BRCA cohort and validated in METABRIC using overall survival as the endpoint. Figure 9(a), 9(c), and 9(d) demonstrate significant prognostic stratification in the TCGA cohort, where patients in the high‐risk group exhibited poorer overall survival as compared to the low‐risk group (log‐rank *p* < 0.001). The model achieved a C‐index of 0.66, indicating a moderate predictive performance. Time‐dependent ROC analysis showed AUC values ranging from 0.65 to 0.77 across selected time points for 60 months, supporting its discriminative ability, (Supporting Figure S8(A)). External validation in the METABRIC cohort confirmed the ability of the model to stratify patients into distinct risk groups (log‐rank *p* < 0.001), shown in Figures 9(b), 9(e), and 9(f). However, predictive performance was reduced (C‐index = 0.56; mean AUC ≈ 0.53), suggesting limited generalizability across cohorts. Risk score distribution analysis showed a clear trend of increased mortality in the high‐risk group. Gene expression heatmaps revealed consistent expression patterns associated with risk stratification (Supporting Figure S9(A)). Age‐stratified analysis indicated that the prognostic significance of the model was more pronounced in patients older than 55 years, while weaker or nonsignificant trends were observed in younger subgroups, likely due to limited sample size and biological heterogeneity (Supporting Figures S9(B)). Calibration analysis demonstrated good agreement between the predicted and observed survival probabilities in the TCGA cohort, whereas deviations were observed in the METABRIC dataset, indicating cohort‐specific variability (Supporting Figure S8(B)).

FIGURE 9Prognostic performance of the five‐gene signature in TCGA and METABRIC cohorts. Kaplan–Meier survival curves showing overall survival differences between high‐ and low‐risk groups in (a) TCGA and (b) METABRIC cohorts. (c) TCGA risk score distribution. (d) TCGA survival status. (e) METABRIC risk score distribution. (f) METABRIC survival status. Both TCGA and METABRIC cohorts illustrate increased mortality in the high‐risk group.

**Figure:**
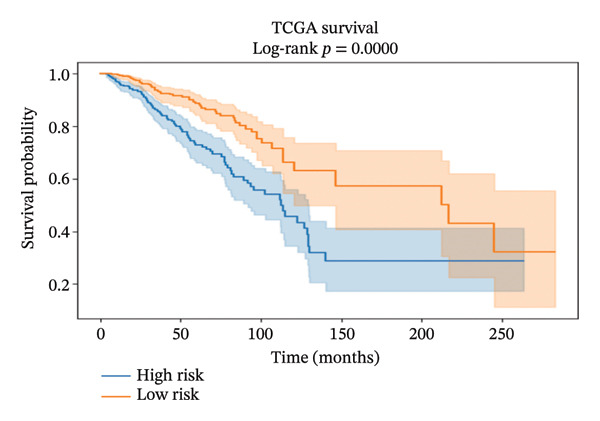
(a)

**Figure:**
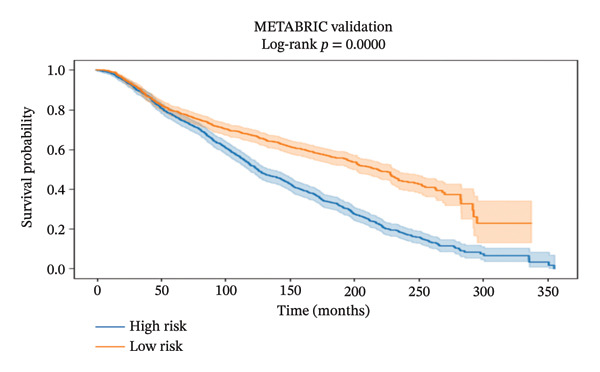
(b)

**Figure:**
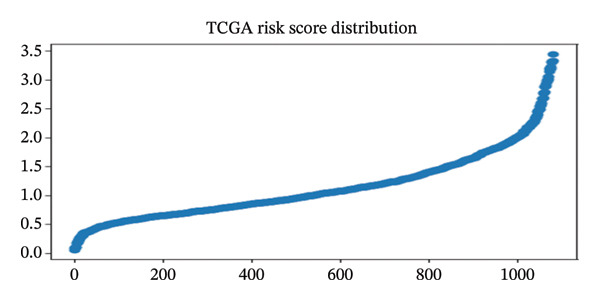
(c)

**Figure:**
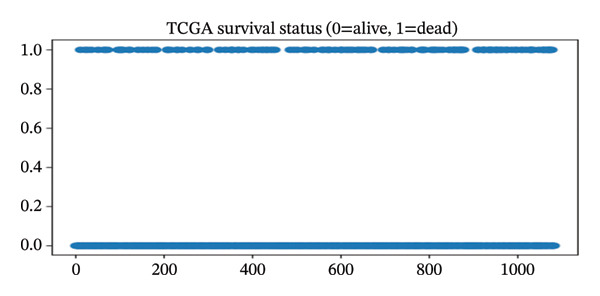
(d)

**Figure:**
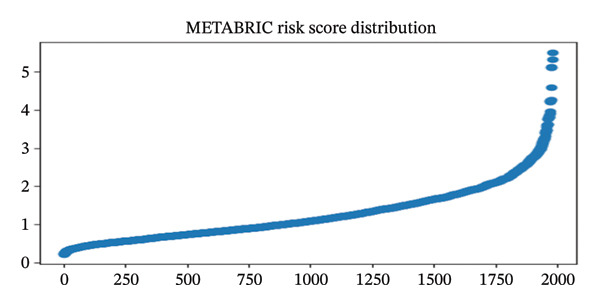
(e)

**Figure:**
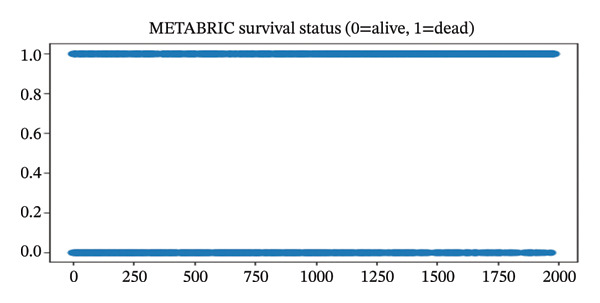
(f)

## 4. Discussion

This study explores how a structurally unique lipid derived from *A. muciniphila* shapes systemic IRs and connects to BRCA‐associated transcriptional programs. By integrating donor‐adjusted PBMC transcriptomics with immune deconvolution, tumor immune infiltration profiling, and survival modeling, the analysis proposes a system‐level link between microbiota‐derived lipid signaling and the breast tumor immune microenvironment.

The reanalysis of the PBMC RNA‐seq data with donor included as a covariate confirmed that the main drivers of transcriptional variation were treatment condition and exposure time rather than interindividual differences. PCA and hierarchical clustering of variance‐stabilized expression profiles showed that samples were grouped predominantly according to stimulation and time point, with donor effects contributing only to the secondary structure. These findings indicate that the DEGs captured in the study largely reflect lipid‐induced immune modulation and are not artifacts of donor‐specific baseline variation.

The time‐resolved DEG patterns revealed an early suppressive response followed by a later activation phase after exposure to the *A. muciniphila*‐derived lipid. At 2 h, downregulated genes were more frequent, whereas 6 and 12 h were characterized by progressively increasing numbers of upregulated transcripts and higher‐amplitude fold changes. This temporal switch suggests that microbial‐derived lipids do not simply impose a static immune state but instead reprogram PBMCs through a biphasic response, in which initial dampening may be followed by the engagement of compensatory or adaptive immune pathways that increasingly overlap with BRCA‐associated gene signatures.

The overlap between lipid‐responsive DEGs in PBMCs and BRCA‐associated genes, combined with immune‐related filtering, supports a mechanistic interface between systemic immune signaling and breast tumor biology. A focused subset of 30 immune‐related genes, derived from intersecting the PBMC DEGs with TISIDB BRCA immune genes, was enriched in pathways linked to adipocytokine signaling, AMPK activation, pluripotency, and peptide hormone metabolism. These pathways sit at the intersection of metabolism, stress responses, and immune regulation and are well‐recognized contributors to cancer initiation and progression. Thus, the transcriptional footprint of the *A. muciniphila* lipid in circulating immune cells recapitulates modules that are also dysregulated in the breast tumor context, arguing for a shared regulatory landscape rather than a coincidental overlap.

The immune deconvolution analysis of PBMC transcriptomes provides functional support for this interpretation. CIBERSORT‐based deconvolution showed that lipid‐associated stimuli induce measurable shifts in immune cell composition, particularly affecting macrophage polarization and cytotoxic effector populations. LPS stimulation drove a strong increase in M1 macrophages, whereas Pam3CSK4 and FSL1 were associated with reduced fractions of activated NK cells and CD8^+^ T cells. M2 macrophages remained at relatively low levels overall, and Tregs showed only modest, nondirectional fluctuations. These patterns indicate that microbial lipid‐related stimuli can remodel systemic immunity, balance proinflammatory and regulatory signals, and adjust the cytotoxic arm of the IR. Such systemic remodeling is consistent with a scenario in which circulating immune cells function as interpreters of microbial cues that ultimately influence tumor immune surveillance.

The tumor‐side analyses in TCGA‐BRCA further refine this picture by linking the PBMC‐derived hub genes to immune infiltration patterns within breast tumors. ADIPOR1, ALDH1A1, KLF4, MYC, and CXCL10 emerged as central regulators in network analyses, occupying highly connected positions within the PPI and ceRNA networks. Immune infiltration correlations in TCGA‐BRCA revealed that these genes associate with distinct but partially overlapping immune cell signatures. ADIPOR1 and KLF4 showed positive correlations with M2 macrophages and resting CD4^+^ memory T cells, and negative associations with M0 macrophages, activated NK cells, CD8^+^ T cells, and Tregs, consistent with a predominantly immunosuppressive or tolerogenic profile. In contrast, CXCL10 and MYC were positively linked to M1 macrophages, activated CD4^+^ and CD8^+^ T cells, and NK cells, and negatively correlated with M2 macrophages and Tregs, reflecting a more immune‐activating phenotype. ALDH1A1 displayed a mixed pattern, associating with both effector and regulatory subsets. Collectively, these results suggest that the same hub genes perturbed by microbiota‐derived lipid exposure in PBMCs are also embedded within tumor immune circuits that balance macrophage polarization and T‐cell activity.

The integration of noncoding RNAs into this framework adds another layer of regulation. By restricting the ceRNA network to ENCORI‐supported interactions that exhibit significant negative correlations, the analysis focuses on miRNA–mRNA and miRNA–lncRNA relationships that are more likely to represent functional repression rather than incidental coexpression. Within this refined network, several miRNAs with established oncogenic roles in BRCA, such as miR‐130b‐3p, miR‐454‐3p, and miR‐301b‐3p, were upregulated and negatively correlated with key hub genes including KLF4, ALDH1A1, and ADIPOR1. Conversely, tumor‐suppressive miRNAs such as miR‐34c‐5p, miR‐29 family members, and miR‐195‐5p showed downregulation. LncRNAs such as FGD5‐AS1 and OTUD6B‐AS1 emerged as potential modulators of chemoresistance and radioresistance. These regulatory axes offer a plausible route by which microbial lipid‐driven perturbations in PBMCs could propagate through RNA networks to reshape gene expression programs relevant to BRCA growth, immune evasion, and treatment response.

Genomic profiling of *A. muciniphila* supports the translational relevance of these interactions by demonstrating a favorable safety and functional profile. The absence of detectable ARGs, VFs, or plasmids, combined with enrichment in vitamin biosynthesis, amino acid metabolism, exopolysaccharide production, and SCFA pathways, is consistent with emerging evidence that *A. muciniphila* can modulate systemic immunity and metabolic homeostasis without overt pathogenic risk. These features strengthen the rationale for considering *A. muciniphila*‐derived components, including the a15:0‐i15:0 PE lipid, as candidate adjuncts for immune‐focused therapies rather than as direct cytotoxic agents.

The prognostic modeling adds a complementary clinical dimension. A five‐gene expression signature derived from the network analysis stratified patients in the TCGA‐BRCA cohort into distinct risk groups with moderate predictive performance, and maintained its prognostic association in the independent METABRIC cohort despite reduced discrimination. The observed attenuation of performance in METABRIC likely reflects differences in cohort composition, treatment histories, and transcriptomic platforms, emphasizing the need for further validation in larger and clinically homogeneous populations. The stronger prognostic impact in older patients suggests age‐dependent or menopausal status‐linked heterogeneity in how these immune metabolic genes integrate into tumor progression, which is particularly relevant as the study highlights the perimenopausal age window as a clinically underexplored subgroup in microbiome–cancer research.

Taken together, these findings support a working model in which *A. muciniphila*‐derived lipid signaling induces a structured reprogramming of PBMC transcriptional states and immune cell composition. This systemic reprogramming converges on gene and pathway modules that are also active in the breast tumor microenvironment, particularly those governing macrophage polarization, cytotoxic T‐cell activity, and noncoding RNA‐mediated regulation of oncogenic and metabolic pathways. In this model, microbial lipids do not act directly on tumor cells but instead shape the immune “soil” in which tumors reside, with context‐dependent consequences, which may either restrain or promote tumor progression depending on the balance of effector and regulatory responses.

However, several limitations must temper the interpretation of these results. The primary transcriptomic dataset is derived from PBMCs of only two donors, and although donor‐adjusted modeling and rigorous QC mitigate interindividual confounding, the sample size remains modest. The study relies on retrospective secondary data without direct measurement of *A. muciniphila* abundance or lipid levels in BRCA patients, and the linkage between PBMC transcriptional responses and tumor immune architecture is inferred through cross‐cohort integration rather than proven experimentally. The ceRNA interactions, while correlation‐filtered and grounded in large‐scale datasets, remain predictive and require functional validation in appropriate cell and animal models. Furthermore, the prognostic model has been evaluated in only two major cohorts, and its utility in specific clinical settings, including immunotherapy response prediction, remains to be tested.

Future work should experimentally validate the proposed lipid–immune–tumor axis using in vitro and in vivo BRCA models exposed to *A. muciniphila*‐derived lipids, with targeted perturbation of the five hub genes and key ncRNAs. Longitudinal clinical studies integrating microbiome profiling, targeted metabolomics, and deep immune phenotyping in BRCA patients are needed to track how microbial lipid signaling and ceRNA networks evolve across stages and therapies. Finally, testing combinatorial strategies that pair probiotics or postbiotics with immune checkpoint inhibitors or RNA‐based therapeutics could clarify whether microbiota‐informed interventions can enhance response in specific age‐ and subtype‐defined patient groups.

## 5. Limitations

• This study is limited by the absence of donor clinical metadata such as age and sex, which were not provided in the original GEO submission.
• All analyses are based on computational methods (multiomics integration, ceRNA network construction, and immune infiltration analysis) without experimental validation in vitro or in vivo.
• The genomic characterization of *A. muciniphila* supports its probiotic potential; however, the functional effects of its lipid metabolites on BRCA cells or tumor–immune interactions were not directly tested.
• The focus on the perimenopausal cohort (45–55 years) may limit the applicability of these findings to other populations.
• It is important to note that this study does not directly investigate the gut–breast axis. The analyses are based on transcriptomic data derived from PBMCs rather than breast tumor tissues.
• Limited availability of PBMC datasets with microbiota‐derived interventions.
• Reduced generalizability of the five‐gene model across cohorts and age groups.

## 6. Conclusion

This study delineates how a structurally distinct lipid derived from *A. muciniphila* can reprogram circulating immune cells and converge on transcriptional and immune pathways that are active in BRCA. By combining donor‐adjusted PBMC transcriptomics, immune deconvolution, ceRNA network analysis, and survival modeling, this work supports a model in which microbial lipid signaling shapes macrophage polarization, cytotoxic T‐cell activity, and noncoding RNA‐mediated regulation of key immune–oncogenic genes such as ADIPOR1, ALDH1A1, KLF4, MYC, and CXCL10. The resulting five‐gene signature demonstrated prognostic value across independent cohorts, particularly in older patients, highlighting its potential as a clinically relevant biomarker panel. While the analyses are correlative and based on retrospective data, they provide a coherent system‐level framework linking gut‐derived metabolites, systemic immune remodeling, and the breast tumor immune microenvironment, and they lay the groundwork for future mechanistic and translational studies.

**Nomenclature**
DE‐mRNA: Differentially expressed mRNA
ncRNA: Noncoding RNA
hsa‐mir: *Homo sapiens* miRNA
lncRNA: Long noncoding RNA

## Author Contributions

Uma Chaudhary and Arya A. S. worked on conceptualization, data curation, formal analysis, and writing–original draft, review, and editing. Mythili A. supervised writing, conceptualization, and review. Uma Chaudhary and Arya A. S. contributed equally to this work and share first authorship.

## Funding

Open access funding was provided by the Vellore Institute of Technology. No funding was received for conducting this study.

## Disclosure

All authors have read and approved the final manuscript.

## Ethics Statement

This study used publicly available GEO data (GSE199367) and did not involve human participants, animal experiments, or clinical samples; therefore, institutional ethical approval was not required.

## Consent

Consent is not required as no individual personal data are included in this manuscript and the study did not involve human participants or animal subjects.

## Conflicts of Interest

The authors declare no conflicts of interest.

## Supporting Information

Additional supporting information can be found online in the Supporting Information section.

## Supporting information


**Supporting Information** Supporting Figure S1: Topological network analysis of MCODE‐derived hub gene clusters using CytoHubba in Cytoscape, highlighting key central genes. Supporting Figure S2: Top 10 highly connected cliques identified from MCODE clusters using the MCLique plugin in Cytoscape. Supporting Figure S3: Heatmaps showing partial correlations between hub genes and immune cell fractions (CD4+ T cells, CD8+ T cells, macrophages, Tregs, and NK cells) in BRCA samples estimated by CIBERSORT. Supporting Figure S4: Scatter plots depicting negative correlations between KLF4, ALDH1A1, or ADIPOR1 and their regulatory miRNAs in BRCA from the ENCORI database. Supporting Figure S5: TIMER‐based heatmap of purity‐adjusted partial Spearman correlations among the five signature genes (ADIPOR1, CXCL10, KLF4, MYC, and ALDH1A1) in breast cancer. Supporting Figure S6: TIMER Gene_DE boxplots comparing expression of ADIPOR1, CXCL10, ALDH1A1, KLF4, and MYC in BRCA tumor versus normal tissues. Supporting Figure S7: Oncoprint summarizing mutation and copy number alterations of ADIPOR1, CXCL10, ALDH1A1, MYC, and KLF4 across breast cancer samples. Supporting Figure S8: Time‐dependent ROC and calibration curves evaluating discrimination and calibration of the five‐gene prognostic model in TCGA and METABRIC cohorts. Supporting Figure S9: Heatmap of the five‐gene expression ordered by the risk score and Kaplan–Meier curves for age‐stratified subgroups, showing risk group survival differences by age. Supporting Table S1: Baseline clinical and sample characteristics for the GSE199367 cohort. Supporting Table S2: Top 10 CytoHubba‐ranked hub genes across 12 network topological algorithms. Supporting Table S3: ULCAN‐based summary of regulation patterns (up/down) of genes, miRNAs, lncRNAs, and circRNAs across cancer stages I–IV. Supporting Table S4: ENCORI‐derived correlations between hub genes, miRNAs, and lncRNA in BRCA, including significant interaction pairs with *p* values and Pearson r.

## Data Availability

The authors confirm that the data supporting the findings of this study are available within the article, and its supplementary and publicly available data sources have been mentioned in the manuscript. Any further data will be made available upon reasonable request. GSE199367 dataset‐https://www.ncbi.nlm.nih.gov/geo/query/acc.cgi?acc=GSE199367 GEPIA‐https://gepia.cancer-pku.cn/ Probiominiserver‐https://probiomindb.imst.nsysu.edu.tw/ eggNOG‐mapper v2‐https://eggnog-mapper.embl.de/ KEGG REST API‐https://www.kegg.jp/kegg/rest/keggapi.html InteractiVenn‐https://www.interactivenn.net/ TISIDB‐https://cis.hku.hk/TISIDB/index.php bc‐GenExMiner v5.1‐https://bcgenex.ico.unicancer.fr/BC-GEM/GEM-Requete.php?mode=11 ENCORI‐https://starbase.sysu.edu.cn/ miRNet 2.0‐https://www.mirnet.ca/ mirDIP4.1‐https://ophid.utoronto.ca/mirDIP/ miRTarBase‐https://mirtarbase.cuhk.edu.cn/%7EmiRTarBase/miRTarBase_2025/php/index.php LncExpDB‐https://ngdc.cncb.ac.cn/lncexpdb/ piRNAQuest V.2‐https://dibresources.jcbose.ac.in/zhumur/pirnaquest2/start.php piRNAdb‐https://www.pirnadb.org/ UALCAN‐https://ualcan.path.uab.edu/cgi-bin/ualcan-res.pl Oncolnc‐https://www.oncolnc.org/ cBioPortal‐https://www.cbioportal.org/ EnrichR‐https://maayanlab.cloud/Enrichr/ STRING‐https://string-db.org/ TCSBN‐https://inetmodels.com/ The Human Protein Atlas‐https://www.proteinatlas.org/ TCGA‐BRCA and METABRIC dataset‐https://www.cbioportal.org/datasets
