# Supplementary material for: Akkermansia muciniphila–Driven ceRNA Networks Regulate Immune Modulation and Breast Cancer Progression
Source: Breast J. 2026 May 26;2026:1416629. doi: 10.1155/tbj/1416629 (PMC13202727; doi:10.1155/tbj/1416629)
Supplement: Supplementary file 1 — Supporting Information Supporting Figure S1: Topological network analysis of MCODE‐derived hub gene clusters using CytoHubba in Cytoscape, highlighting key central genes. Supporting Figure S2: Top 10 highly connected cliques identified from MCODE clusters using the MCLique plugin in Cytoscape. Supporting Figure S3: Heatmaps showing partial correlations between hub genes and immune cell fractions (CD4+ T cells, CD8+ T cells, macrophages, Tregs, and NK cells) in BRCA samples estimated by CIBERSORT. Supporting Figure S4: Scatter plots depicting negative correlations between KLF4, ALDH1A1, or ADIPOR1 and their regulatory miRNAs in BRCA from the ENCORI database. Supporting Figure S5: TIMER‐based heatmap of purity‐adjusted partial Spearman correlations among the five signature genes (ADIPOR1, CXCL10, KLF4, MYC, and ALDH1A1) in breast cancer. Supporting Figure S6: TIMER Gene_DE boxplots comparing expression of ADIPOR1, CXCL10, ALDH1A1, KLF4, and MYC in BRCA tumor versus normal tissues. Supporting Figure S7: Oncoprint summarizing mutation and copy number alterations of ADIPOR1, CXCL10, ALDH1A1, MYC, and KLF4 across breast cancer samples. Supporting Figure S8: Time‐dependent ROC and calibration curves evaluating discrimination and calibration of the five‐gene prognostic model in TCGA and METABRIC cohorts. Supporting Figure S9: Heatmap of the five‐gene expression ordered by the risk score and Kaplan–Meier curves for age‐stratified subgroups, showing risk group survival differences by age. Supporting Table S1: Baseline clinical and sample characteristics for the GSE199367 cohort. Supporting Table S2: Top 10 CytoHubba‐ranked hub genes across 12 network topological algorithms. Supporting Table S3: ULCAN‐based summary of regulation patterns (up/down) of genes, miRNAs, lncRNAs, and circRNAs across cancer stages I–IV. Supporting Table S4: ENCORI‐derived correlations between hub genes, miRNAs, and lncRNA in BRCA, including significant interaction pairs with p values and Pearson r. [file TBJ-2026-1416629-s001.docx]

**Supplementary Figures**


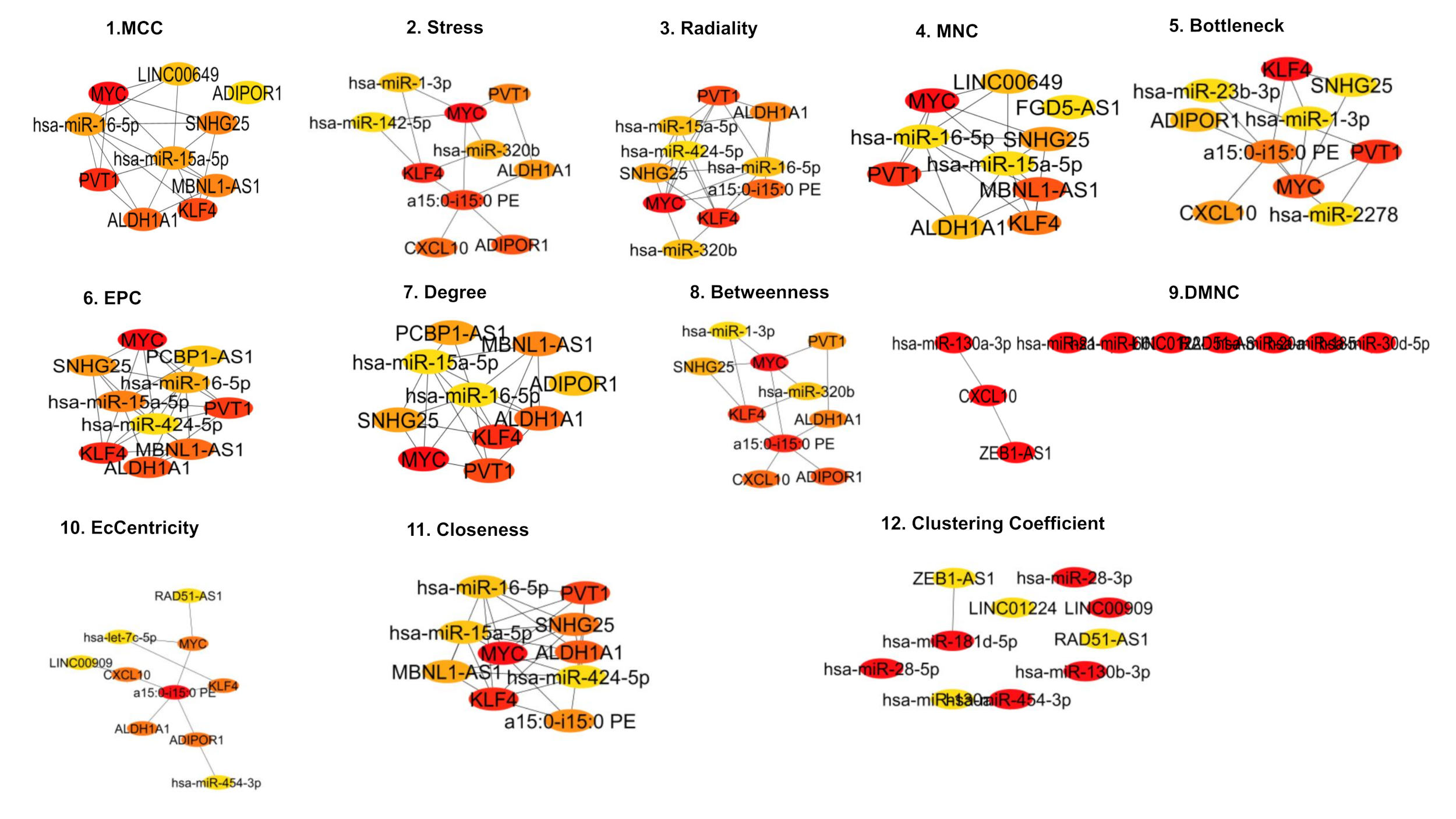


**Fig.1** Topological analysis of hub genes from MCODE-derived clusters using CytoHubba in Cytoscape.


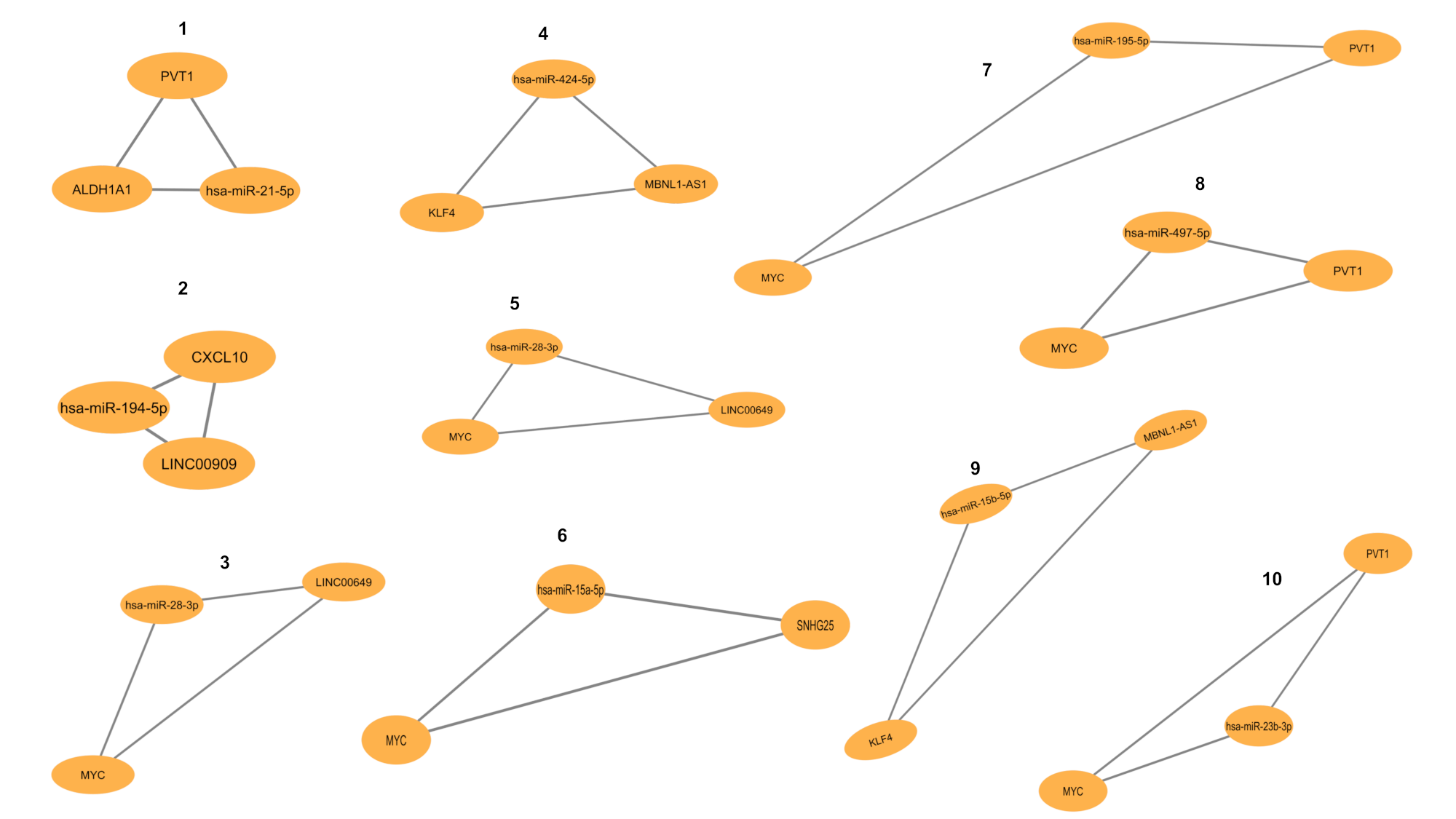


**Fig.2** Top 10 cliques generated from MCODE clusters using the MCLique plugin in Cytoscape.


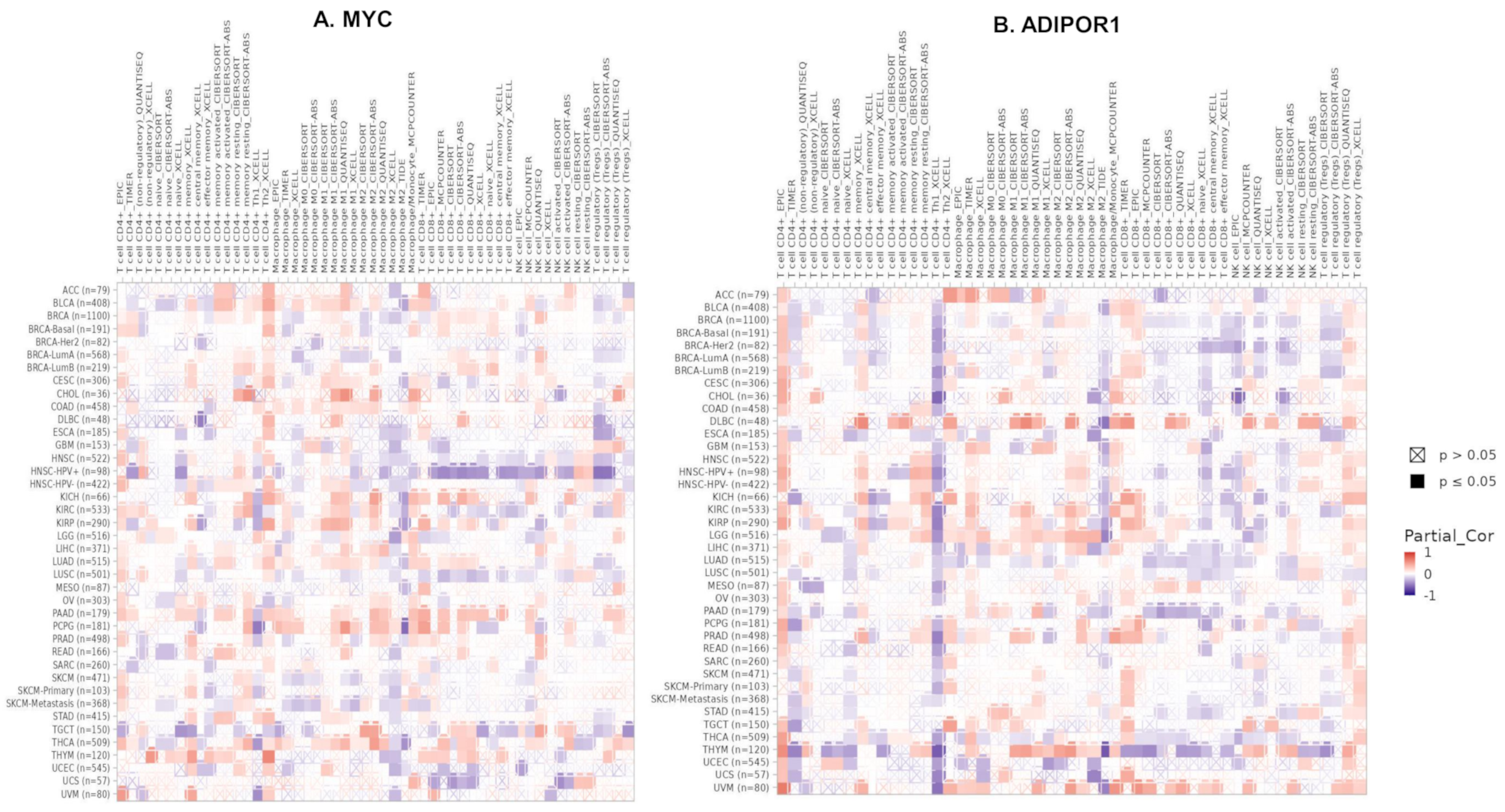


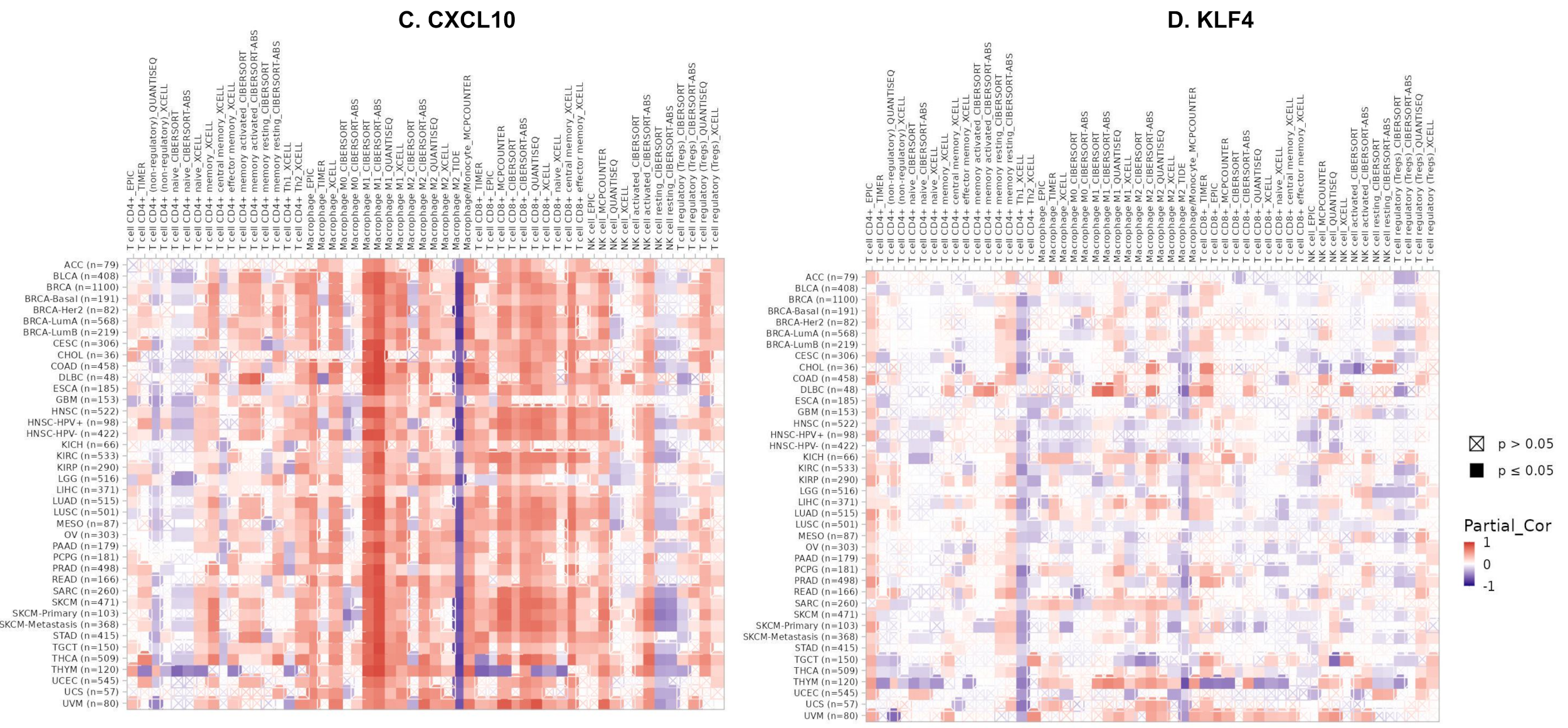


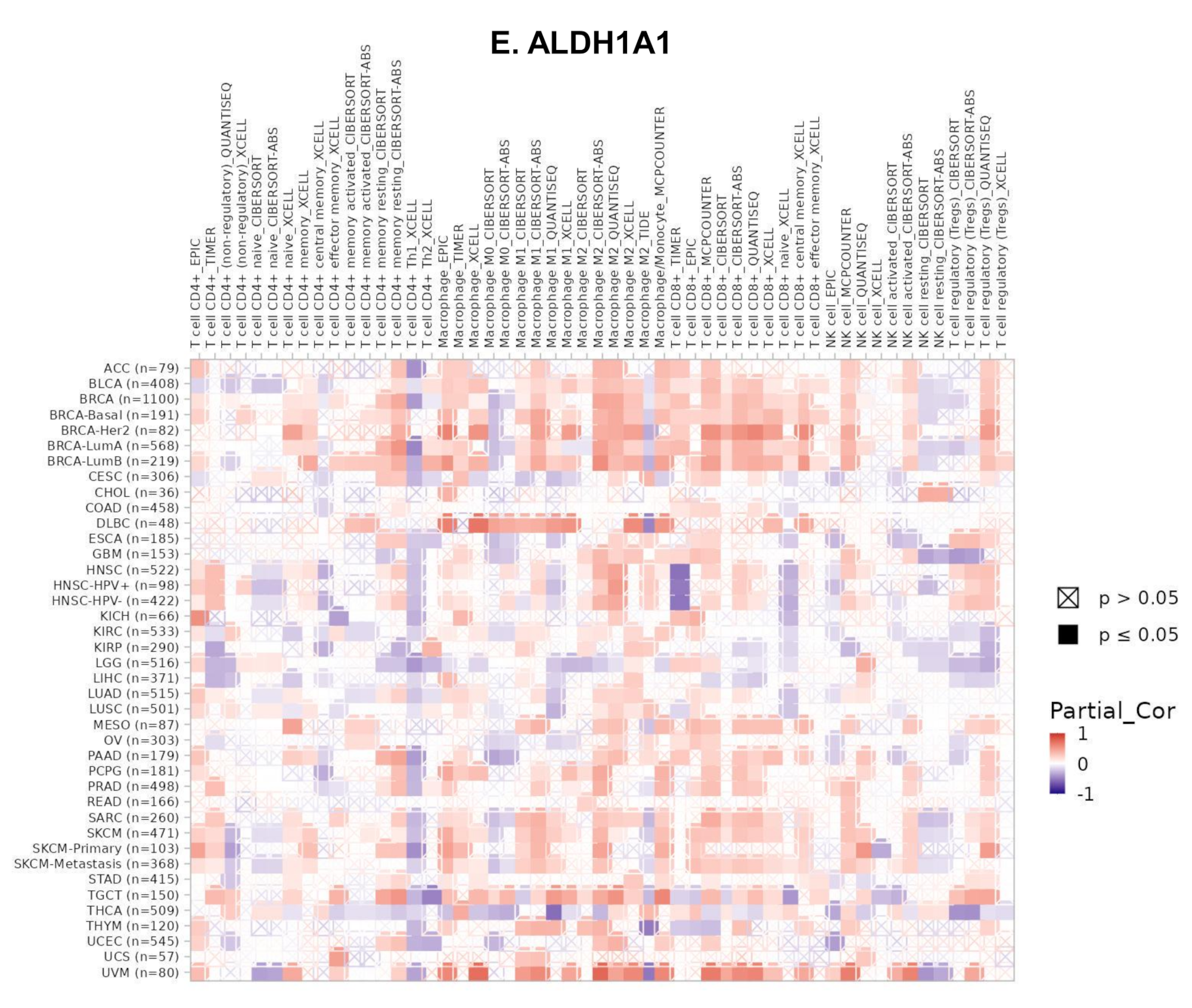


**Fig.3** Immune infiltrate–hub gene association in BRCA. Partial correlations between hub genes and immune cell fractions, including CD4+ T cells, CD8+ T cells, macrophages, Tregs, and NK cells, were analyzed using the CIBERSORT algorithm. Only BRCA samples with $p<0.05$ were retained and shown in the filtered, sorted heatmaps.


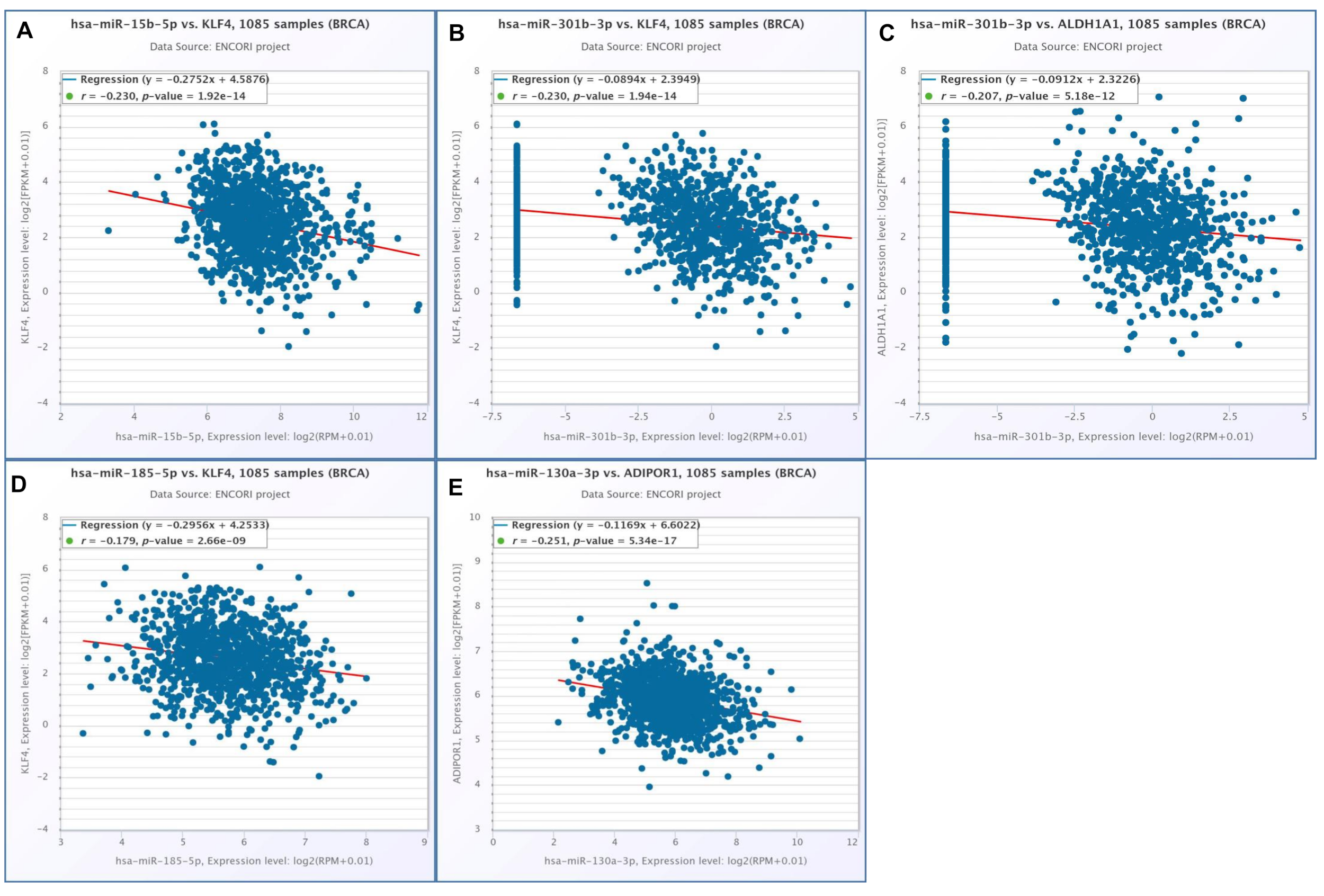


**Fig.4** Correlation of hub genes with regulatory miRNAs in BRCA. Scatter plots show negative correlations between KLF4 or ALDH1A1 or ADIPOR1 expression and the indicated miRNAs across breast cancer samples from the ENCORI database.

**
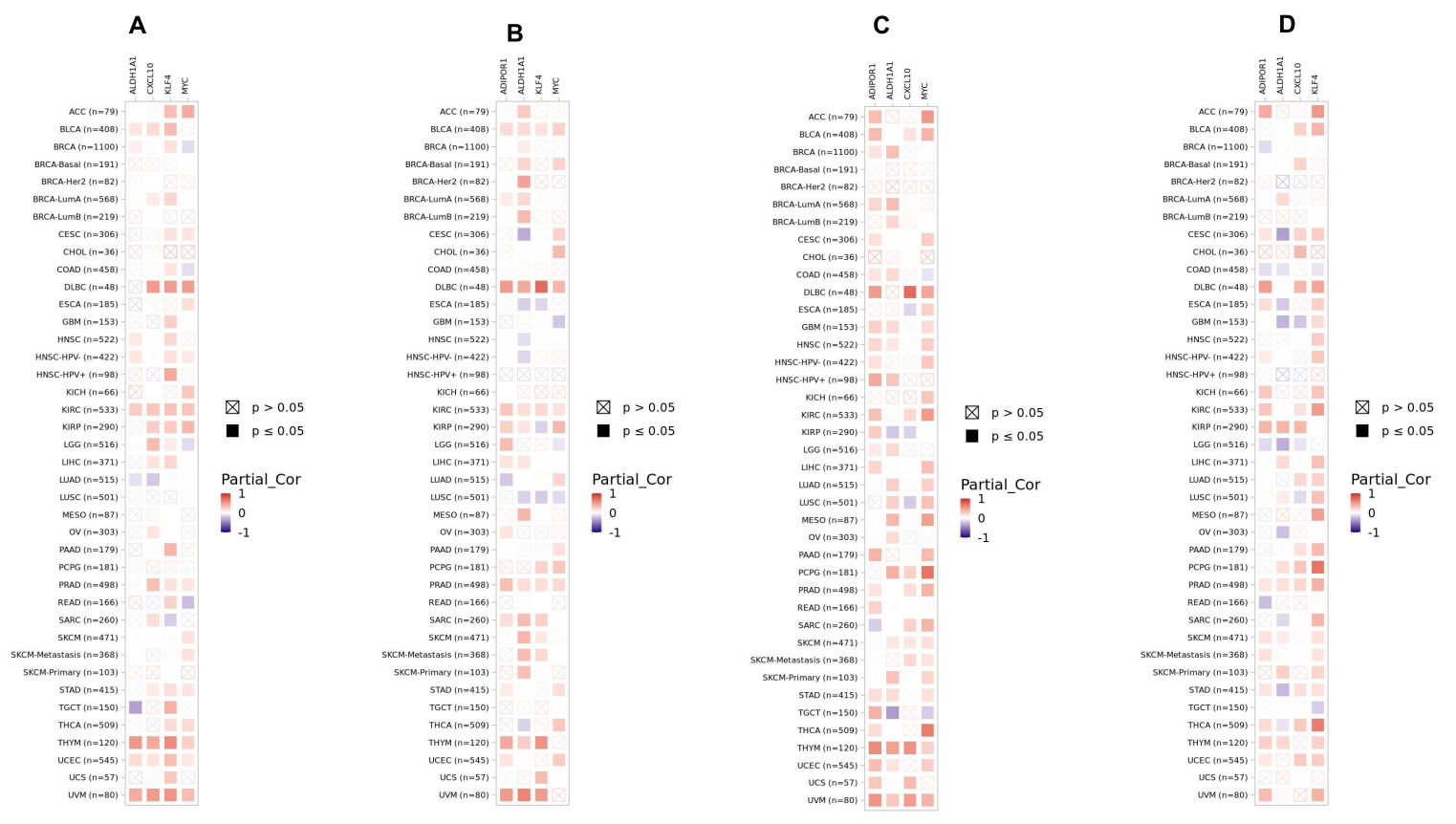
**

**Fig.5** Gene correlation analysis of the five-gene signature in breast cancer using TIMER.

Heatmap represents the purity-adjusted partial Spearman’s correlation coefficients (ρ) among (A) ADIPOR1 (B) CXCL10 (C) KLF4 (D) MYC and (E) ALDH1A1.Red shading denotes positive correlations, while blue shading indicates negative correlations. The analysis highlights heterogeneous correlation patterns.

A.
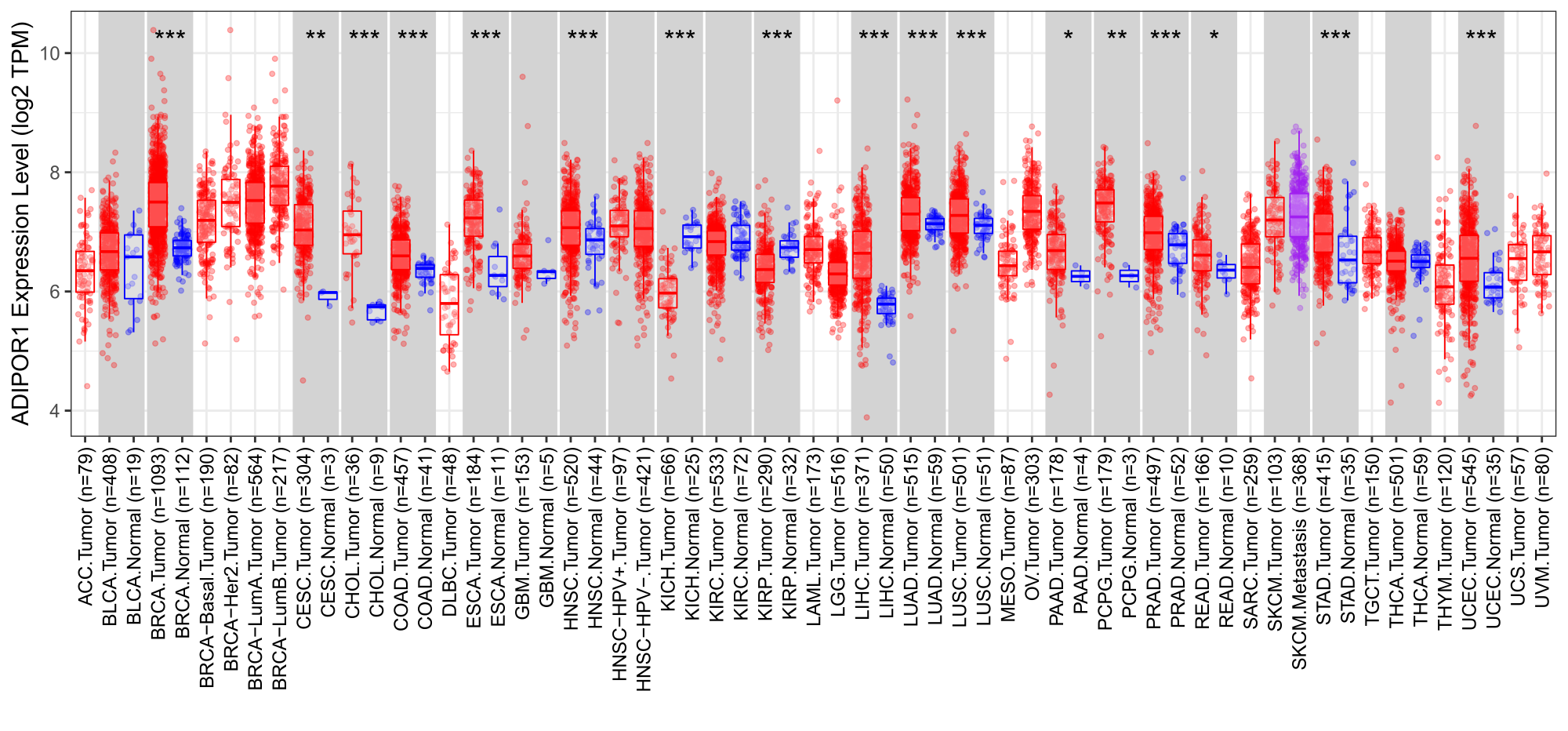


B.
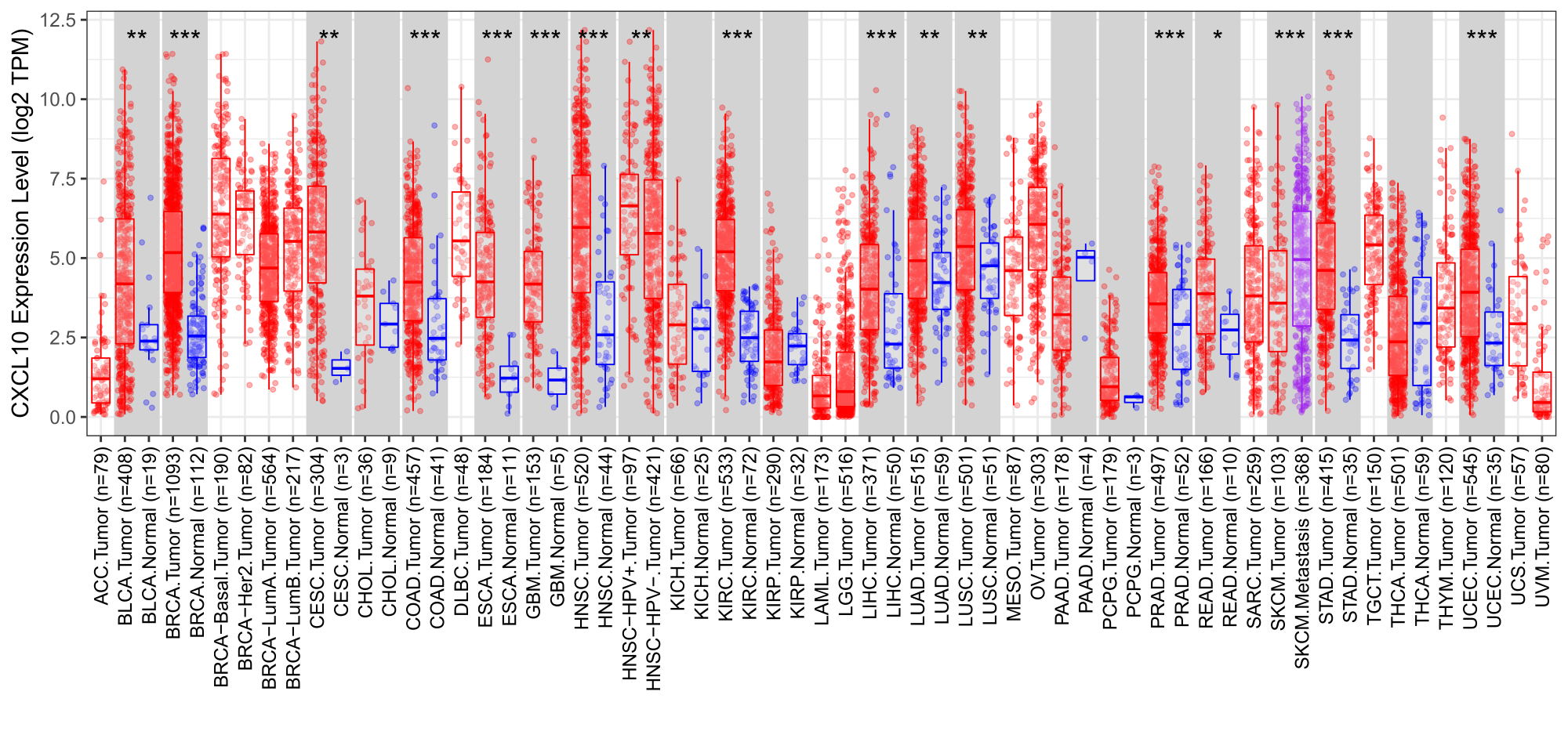


C.
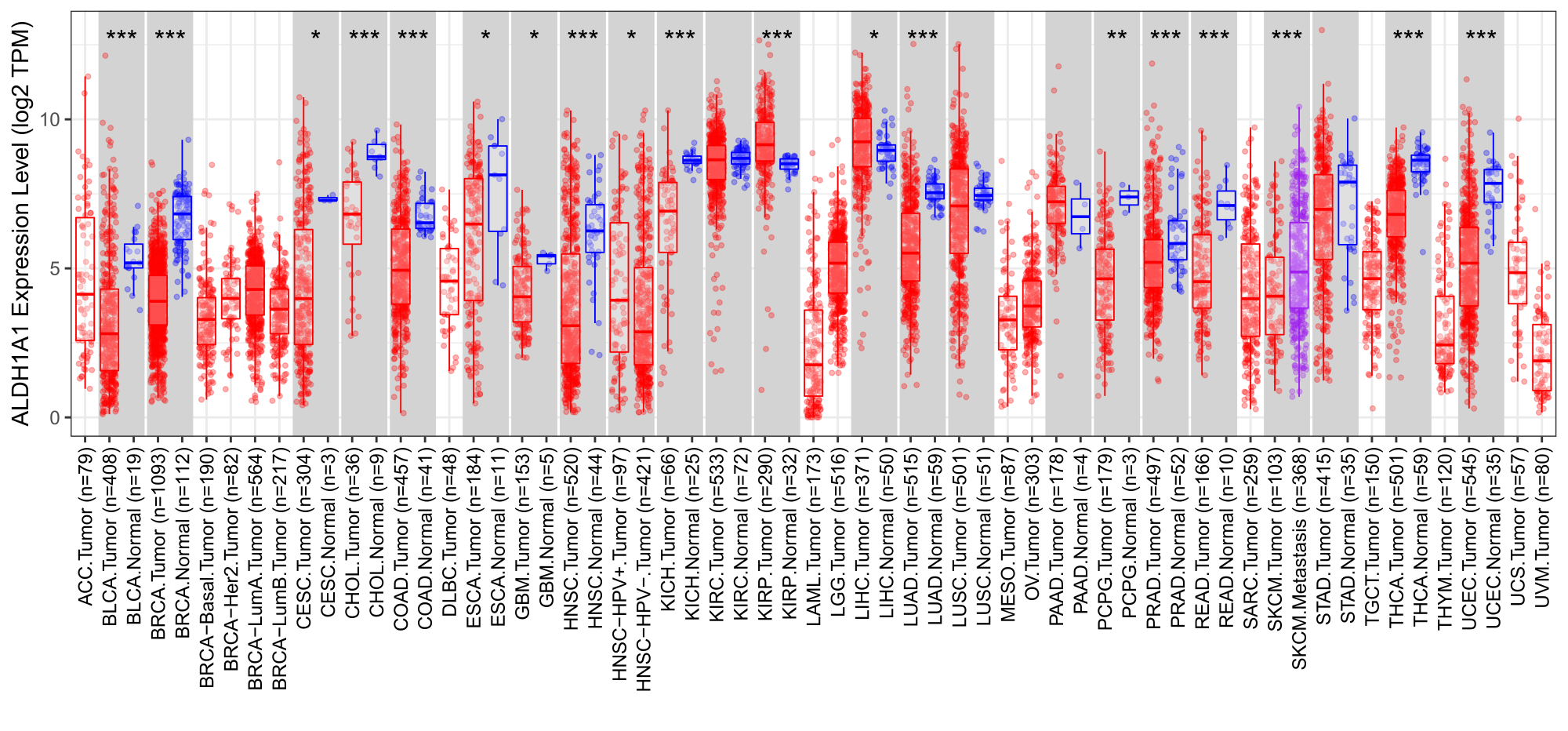


D.
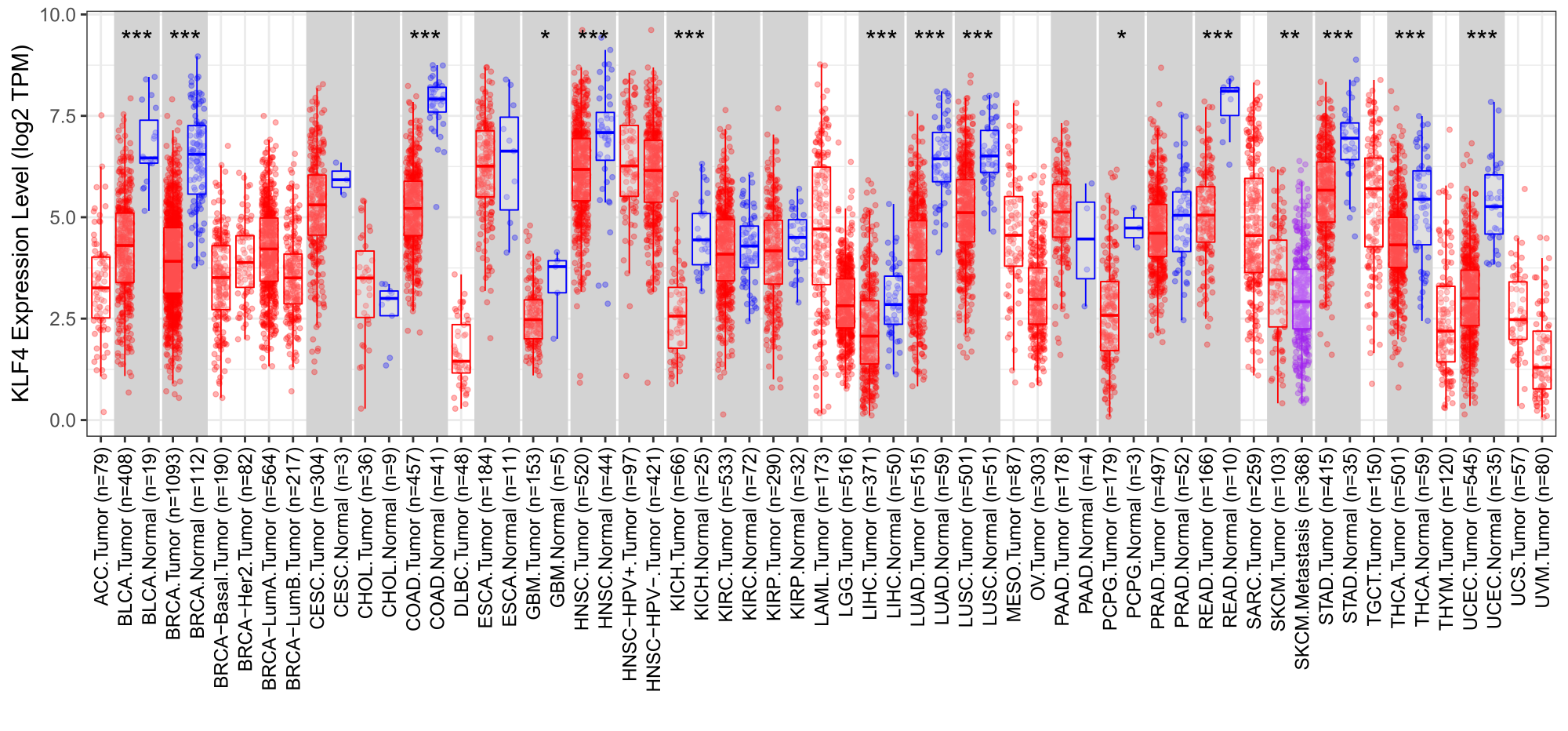


E.
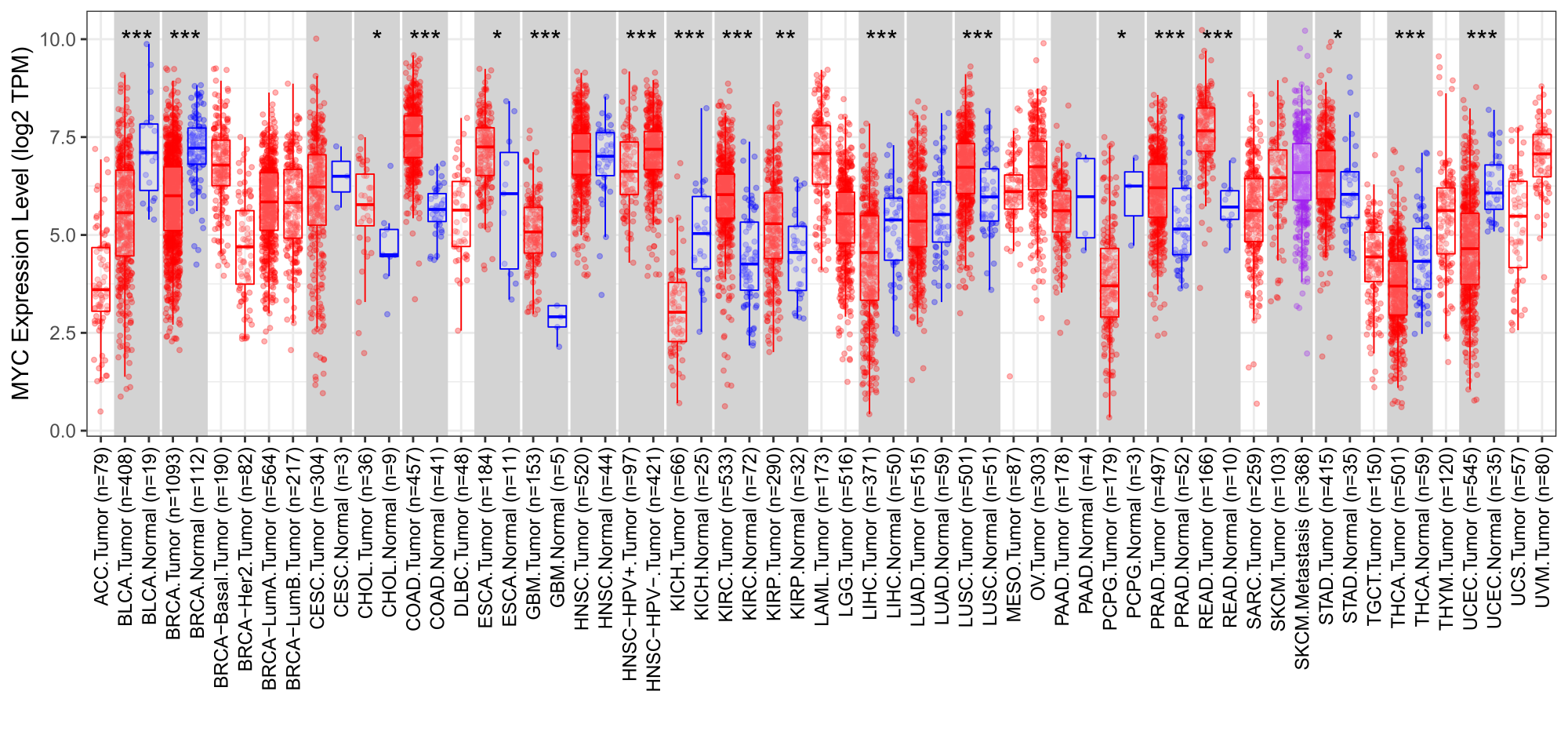


**Fig.6** Differential expression analysis of five candidate genes in breast cancer (BRCA) using TIMER Gene_DE module. Boxplots show gene expression in tumor (red) versus adjacent normal (blue) tissues, with statistical significance assessed by the Wilcoxon test. (A) ADIPOR1 and (B) CXCL10 were significantly upregulated in tumors, while (C) ALDH1A1, (D) KLF4, and (E)MYC were significantly downregulated, highlighting their contrasting roles in breast cancer biology.


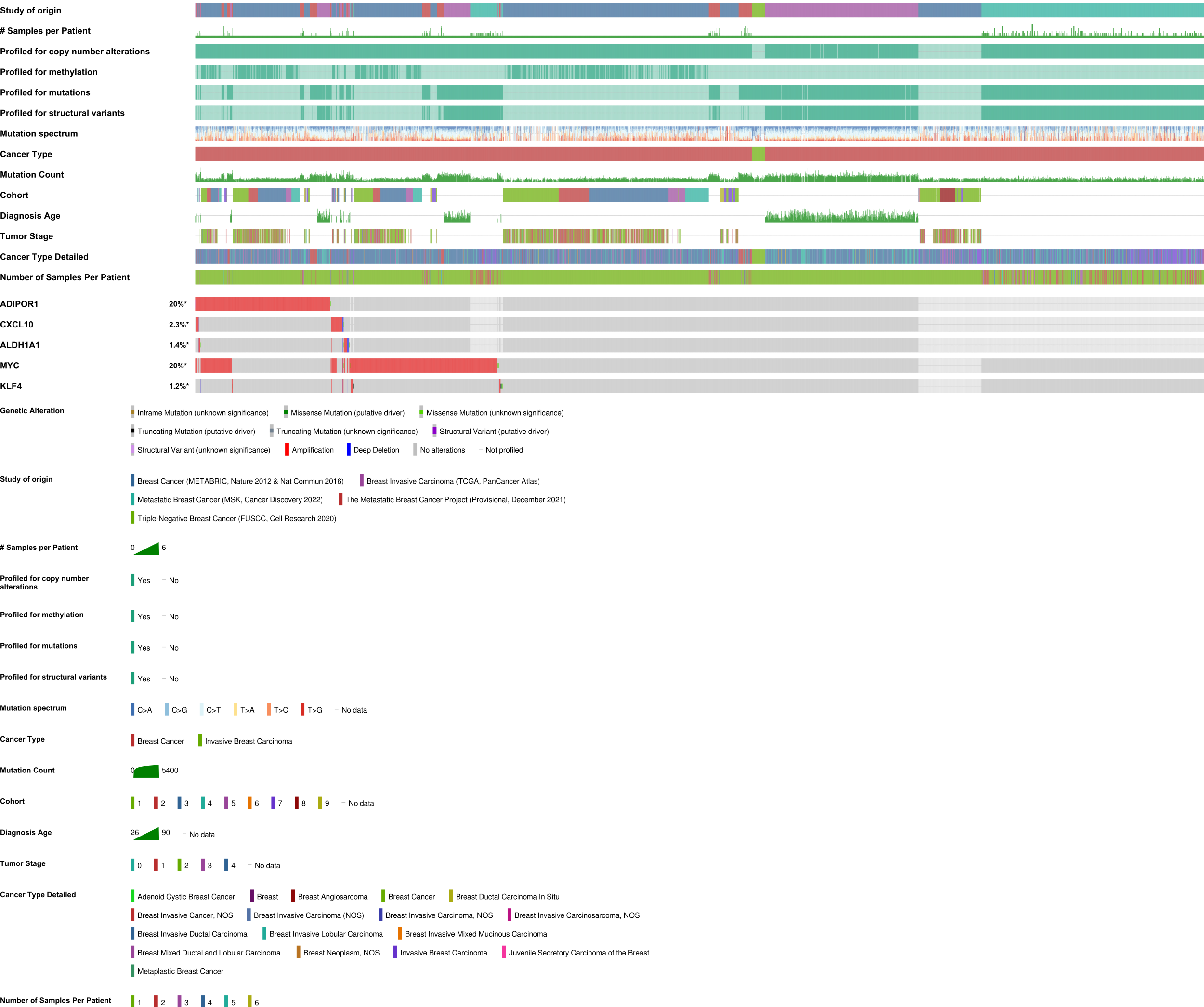


**Fig.7** Oncoprint visualization of genomic alterations in candidate genes across breast cancer samples.
Each column corresponds to an individual patient, and each row represents a queried gene (ADIPOR1, CXCL10, ALDH1A1, MYC, and KLF4). Colored blocks indicate mutation types, copy number alterations, and structural variants, while white blocks indicate no alteration. The right panel summarizes the overall alteration frequency for each gene, highlighting ADIPOR1 (20%) and MYC (20%) as the most frequently altered genes, primarily through amplifications. Alterations were most commonly observed in breast invasive ductal carcinoma cases, underscoring their prevalence in this subtype.


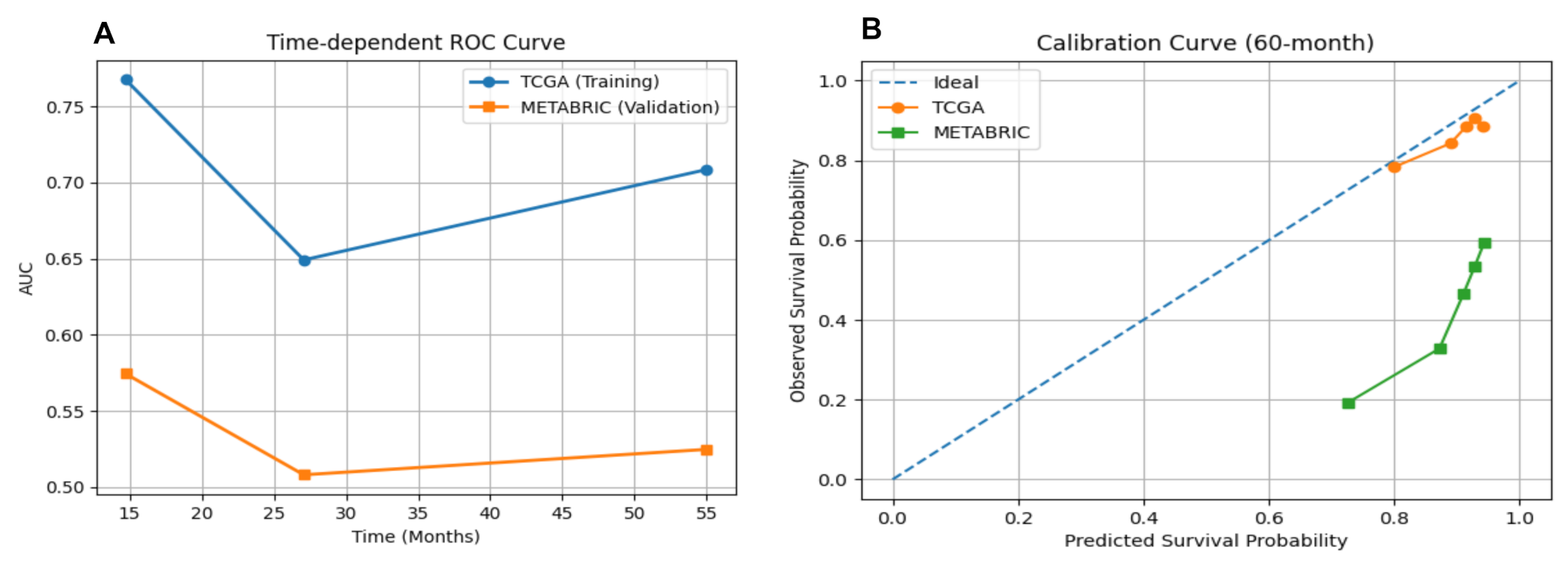


**Fig.8 Predictive performance and calibration of the 5-gene prognostic model.**
(A) Time-dependent ROC curves showing model performance at selected time points in TCGA and METABRIC cohorts. (B) Calibration curves demonstrating agreement between predicted and observed survival probabilities, with better calibration observed in the TCGA cohort compared to METABRIC.


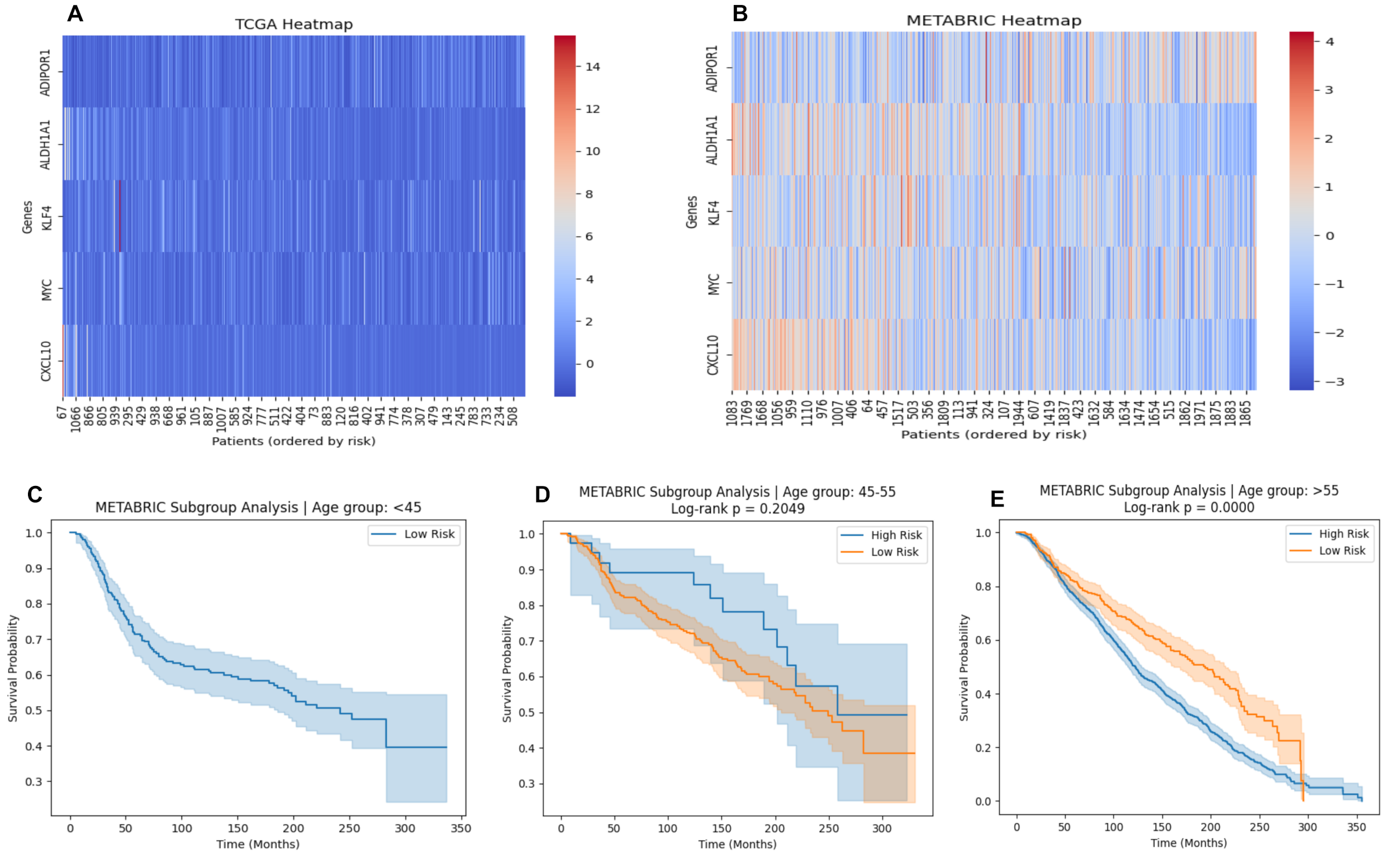


**Fig.9 Expression patterns and subgroup analysis of the 5-gene signature.**
(A) Heatmap showing expression patterns of the five genes across patients ordered by risk score. (B–D) Kaplan–Meier survival curves for age-stratified subgroups (<45, 45–55, >55 years), demonstrating stronger prognostic significance in older patients.

**TABLES**

**Supplementary** **Table 1.** Baseline Characteristics of Samples in GSE199367

| **Sr. No** | **Variable** | **Category** | **Number of Samples** |
| --- | --- | --- | --- |
| 1 | Donor | Donor 1 | 63 |
|  |  | Donor 2 | 63 |
| 2 | Timepoint | 2 hours | 42 |
|  |  | 6 hours | 42 |
|  |  | 12 hours | 42 |
| 3 | Treatment | Stim_A1 | 18 |
|  |  | Stim_A2 | 18 |
|  |  | Stim_A3 | 18 |
|  |  | DMSO (Negative Control) | 18 |
|  |  | LPS (Positive Control) | 18 |
|  |  | Pam3CSK4 | 18 |
|  |  | FSL-1 | 18 |
| 4 | Replicates | Per condition | 3 |
| Total samples = 126 | | | |

**Supplementary Table 2.** Top 10 Cytohubba nodes in ranking order for all 12 topological algorithms.

| **Order of node of top 10 nodes** | **Topological Algorithm** |
| --- | --- |
| **1** | **MCC** |
| 1 | PVT1 |
| 2 | MBNL1-AS1 |
| 3 | hsa-miR-16-5p |
| 4 | LINC00649 |
| 5 | MYC |
| 6 | ALDH1A1 |
| 7 | SNHG25 |
| 8 | ADIPOR1 |
| 9 | KLF4 |
| 10 | hsa-miR-15a-5p |
| **2** | **Degree** |
| 1 | PVT1 |
| 2 | MBNL1-AS1 |
| 3 | hsa-miR-16-5p |
| 4 | PCBP1-AS1 |
| 5 | MYC |
| 6 | ALDH1A1 |
| 7 | SNHG25 |
| 8 | ADIPOR1 |
| 9 | KLF4 |
| 10 | hsa-miR-15a-5p |
| **3** | **Bottleneck** |
| 1 | PVT1 |
| 2 | hsa-miR-23b-3p |
| 3 | hsa-miR-2278 |
| 4 | hsa-miR-1-3p |
| 5 | MYC |
| 6 | SNHG25 |
| 7 | a15:0-i15:0 PE |
| 8 | ADIPOR1 |
| 9 | KLF4 |
| 10 | CXCL10 |
| **4** | **DMNC** |
| 1 | ZEB1-AS1 |
| 2 | hsa-miR-130a-3p |
| 3 | hsa-miR-30d-5p |
| 4 | hsa-miR-185-5p |
| 5 | hsa-miR-20a-5p |
| 6 | RAD51-AS1 |
| 7 | LINC01224 |
| 8 | hsa-miR-663a |
| 9 | CXCL10 |
| 10 | hsa-miR-21-5p |
| **5** | **EPC** |
| 1 | PVT1 |
| 2 | MBNL1-AS1 |
| 3 | hsa-miR-16-5p |
| 4 | PCBP1-AS1 |
| 5 | hsa-miR-424-5p |
| 6 | MYC |
| 7 | ALDH1A1 |
| 8 | SNHG25 |
| 9 | KLF4 |
| 10 | hsa-miR-15a-5p |
| **6** | **MNC** |
| 1 | PVT1 |
| 2 | MBNL1-AS1 |
| 3 | hsa-miR-16-5p |
| 4 | FGD5-AS1 |
| 5 | LINC00649 |
| 6 | MYC |
| 7 | ALDH1A1 |
| 8 | SNHG25 |
| 9 | KLF4 |
| 10 | hsa-miR-15a-5p |
| **7** | **EcCentricity** |
| 1 | hsa-miR-454-3p |
| 2 | hsa-let-7c-5p |
| 3 | LINC00909 |
| 4 | RAD51-AS1 |
| 5 | MYC |
| 6 | ADIPOR1 |
| 7 | a15:0-i15:0 PE |
| 8 | ALDH1A1 |
| 9 | KLF4 |
| 10 | CXCL10 |
| **8** | **Closeness** |
| 1 | PVT1 |
| 2 | MBNL1-AS1 |
| 3 | hsa-miR-16-5p |
| 4 | hsa-miR-424-5p |
| 5 | MYC |
| 6 | ALDH1A1 |
| 7 | SNHG25 |
| 8 | a15:0-i15:0 PE |
| 9 | KLF4 |
| 10 | hsa-miR-15a-5p |
| **9** | **Radiality** |
| 1 | PVT1 |
| 2 | hsa-miR-16-5p |
| 3 | MYC |
| 4 | hsa-miR-424-5p |
| 5 | SNHG25 |
| 6 | a15:0-i15:0 PE |
| 7 | ALDH1A1 |
| 8 | hsa-miR-320b |
| 9 | KLF4 |
| 10 | hsa-miR-15a-5p |
| **10** | **Betweenness** |
| 1 | PVT1 |
| 2 | hsa-miR-1-3p |
| 3 | MYC |
| 4 | SNHG25 |
| 5 | a15:0-i15:0 PE |
| 6 | ALDH1A1 |
| 7 | ADIPOR1 |
| 8 | KLF4 |
| 9 | hsa-miR-320b |
| 10 | CXCL10 |
| **11** | **Stress** |
| 1 | PVT1 |
| 2 | hsa-miR-1-3p |
| 3 | MYC |
| 4 | a15:0-i15:0 PE |
| 5 | ALDH1A1 |
| 6 | ADIPOR1 |
| 7 | hsa-miR-142-5p |
| 8 | KLF4 |
| 9 | hsa-miR-320b |
| 10 | CXCL10 |
| **12** | **Clustering Coefficient** |
| 1 | hsa-miR-454-3p |
| 2 | ZEB1-AS1 |
| 3 | hsa-miR-130a-3p |
| 4 | hsa-miR-28-5p |
| 5 | LINC00909 |
| 6 | hsa-miR-181d-5p |
| 7 | hsa-miR-28-3p |
| 8 | RAD51-AS1 |
| 9 | LINC01224 |
| 10 | hsa-miR-130b-3p |

| **Supplemenatry Table 3**. ULCAN database is utilized to check the regulation patterns of genes, miRNAs, lncRNAs, and circRNAs across different cancer stages (I–IV). UP = Upregulated; DOWN = Downregulated. | | | | | |
| --- | --- | --- | --- | --- | --- |
| **Genes** | **Individual Cancer Stages** | **Patient's Race (Asian)** | **Patient's Age(41-60)** | **Menopause Status (Perimenopause)** | **ULCAN Survival Analysis (p-value)** |
| ADIPOR1 | IV-UP | UP | UP | UP | 0.21 |
| CXCL10 | II-UP | UP | UP | UP | 0.42 |
| ALDH1A1 | IV-DOWN | DOWN | DOWN | DOWN | 0.21 |
| MYC | IV-DOWN | DOWN | DOWN | DOWN | 0.57 |
| KLF4 | IV-DOWN | DOWN | DOWN | DOWN | 0.84 |
| hsa-miR-130b-3p | II-UP | UP | UP | UP | 0.00089 |
| hsa-miR-454-3p | II-UP | UP | UP | UP | 0.0091 |
| hsa-miR-130a-3p | IV-DOWN | DOWN | DOWN | DOWN | 0.988 |
| hsa-miR-181b-5p | III-UP | UP | UP | UP | 0.00059 |
| hsa-miR-186-5p | IV-DOWN | DOWN | DOWN | DOWN | 0.0012 |
| hsa-miR-301a-3p | II-UP | UP | UP | UP | 0.07 |
| hsa-miR-301b-3p | IV-UP | UP | UP | UP | 0.02 |
| hsa-miR-877-5p | II -UP | UP | UP | UP | 0.048 |
| hsa-miR-708-5p | I -UP | UP | UP | UP | 0.072 |
| hsa-miR-29a-3p | IV-DOWN | DOWN | DOWN | DOWN | 0.0058 |
| hsa-miR-29c-3p | IV-UP | DOWN | DOWN | DOWN | 0.014 |
| hsa-miR-15b-5p | II-UP | UP | UP | UP | 0.082 |
| hsa-miR-195-5p | IV-DOWN | DOWN | DOWN | DOWN | 0.015 |
| hsa-miR-424-5p | IV-UP | UP | UP | UP | 0.037 |
| hsa-miR-185-5p | IV-UP | UP | UP | UP | 0.013 |
| hsa-miR-7-5p | II-UP | UP | UP | UP | 0.033 |
| FGD5-AS1 | IV-DOWN | DOWN | DOWN | DOWN | 0.0065 |
| OTUD6B-AS1 | IV-DOWN | DOWN | DOWN | DOWN | 0.046 |

Note:

| SAN= Same as Normal |
| --- |
| NA- Data Not Available |
| Cancer Stages (I,II,III,IV, UP and DOWN) |
| UP - Upregulation OR High-Expression |
| DOWN- Downregulation OR Low- Expression |

**Supplementary Table 4.** Correlation between hub genes, miRNAs, and lncRNA in BRCA (ENCORI), showing significant interaction pairs with corresponding p‑values and Pearson r-values.

| **Shared names** | **Interaction** | **Shared names** | **Correlational values** | |
| --- | --- | --- | --- | --- |
|  |  |  | **p-value** | **r-value** |
| ADIPOR1 | interacts with | hsa-miR-130b-3p | 1.70E-01 | -0.042 |
| ADIPOR1 | interacts with | hsa-miR-454-3p | 8.04E-06 | 0.135 |
| ADIPOR1 | interacts with | hsa-miR-130a-3p | 5.34E-17 | -0.251 |
| ADIPOR1 | interacts with | hsa-miR-181b-5p | 2.18E-02 | -0.07 |
| MYC | interacts with | hsa-miR-195-5p | 6.49E-05 | 0.121 |
| MYC | interacts with | hsa-miR-15b-5p | 2.72E-03 | 0.091 |
| MYC | interacts with | hsa-miR-186-5p | 2.88E-05 | 0.127 |
| MYC | interacts with | hsa-miR-301a-3p | 4.22E-02 | 0.062 |
| MYC | interacts with | hsa-miR-301b-3p | 5.32E-04 | 0.105 |
| MYC | interacts with | hsa-miR-708-5p | 1.02E-02 | -0.078 |
| MYC | interacts with | hsa-miR-877-5p | 4.04E-05 | 0.124 |
| MYC | interacts with | hsa-miR-29a-3p | 8.77E-09 | 0.174 |
| MYC | interacts with | hsa-miR-29c-3p | 2.60E-03 | -0.091 |
| MYC | interacts with | hsa-miR-7-5p | 5.65E-01 | 0.017 |
| MYC | interacts with | hsa-miR-185-5p | 7.85E-02 | 0.053 |
| MYC | interacts with | hsa-miR-424-5p | 2.10E-02 | 0.07 |
| ALDH1A1 | interacts with | hsa-miR-301b-3p | 5.18E-12 | -0.207 |
| ALDH1A1 | interacts with | hsa-miR-195-5p | 1.83E-09 | 0.181 |
| ALDH1A1 | interacts with | hsa-miR-185-5p | 7.37E-06 | -0.136 |
| KLF4 | interacts with | hsa-miR-301b-3p | 1.94E-14 | -0.23 |
| KLF4 | interacts with | hsa-miR-29a-3p | 3.71E-05 | 0.125 |
| KLF4 | interacts with | hsa-miR-29c-3p | 4.20E-09 | 0.177 |
| KLF4 | interacts with | hsa-miR-15b-5p | 1.92E-14 | -0.23 |
| KLF4 | interacts with | hsa-miR-424-5p | 9.07E-02 | -0.05 |
| KLF4 | interacts with | hsa-miR-185-5p | 2.66E-09 | -0.179 |
| KLF4 | interacts with | hsa-miR-7-5p | 7.91E-08 | -0.162 |
| CXCL10 | interacts with | hsa-miR-181b-5p | 2.55E-03 | 0.092 |
| CXCL10 | interacts with | hsa-miR-130a-3p | 4.84E-04 | 0.106 |
| hsa-miR-130a-3p | interacts with | FGD5-AS1 | 9.29E-01 | -0.003 |
| hsa-miR-454-3p | interacts with | FGD5-AS1 | 2.30E-01 | 0.036 |
| hsa-miR-130b-3p | interacts with | FGD5-AS1 | 2.17E-02 | -0.07 |
